# Fibroin: A Multi-Functional Bio-Derived Binder for Lithium–Sulfur Batteries

**DOI:** 10.1021/acssuschemeng.5c01291

**Published:** 2025-08-22

**Authors:** Yiming Guo, Roby Soni, Kofi Coke, James B. Robinson, Francesco Iacoviello, Robert S. Young, Rhodri Jervis, Paul R. Shearing, Thomas S. Miller

**Affiliations:** † Electrochemical Innovation Lab, Department of Chemical Engineering, 4919 University College London, Torrington Place, London WC1E 7JE, U.K.; ‡ Advanced Propulsion Lab, Marshgate, University College London, 7 Sidings Street, London E20 2AE, U.K.; § The Faraday Institution, Quad One, Becquerel Avenue, Harwell Campus, Didcot OX11 ORA, U.K.; ∥ The ZERO Institute, 98956 University of Oxford, Holywell House, Osney Mead, Oxford OX2 0ES, U.K.

**Keywords:** Li–S battery, polysulfide shuttle effect, protein binder, battery recycling, aqueous battery binder

## Abstract

Traditionally, binders such as poly­(vinylidene fluoride) (PVDF) have been used within lithium–sulfur (Li–S) batteries, but these present environmental and recyclability challenges and have little to no impact on the processes that drive degradation in the cell’s chemistry. Ideally, a Li–S battery binder would contribute to the mitigation of the polysulfide shuttle effect and negate the impacts of positive electrode volume expansion while being compatible with aqueous ink preparation and low-energy, low-toxicity recycling processes. In this work, we demonstrate that fibroin, an economical and sustainable biological polymer with an abundance of functional groups, can effectively trap polysulfides while still offering the durability, cyclability, and ease of use offered by the current state-of-the-art binder (PVDF). In Li–S coin and pouch cells, fibroin-based electrodes with a high sulfur loading are shown to offer high capacities and Coulombic efficiencies, which in situ and operando analysis shows is due to its beneficial properties as a binder. Importantly, fibroin’s ability to be denatured under mildly acidic conditions is shown to make electrodes significantly easier to recycle than ones prepared using PVDF, which require energy-intensive mechanical processes for recycling. Hence, overall, this study highlights fibroin as a promising ecofriendly alternative binder that not only enhances Li–S battery performance but also offers significant advantages for sustainability and recyclability.

## Graphical abstract

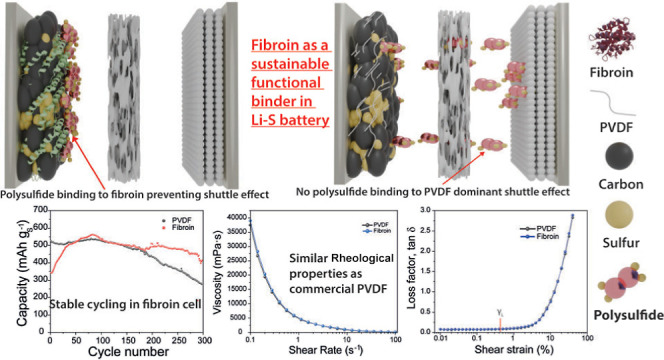

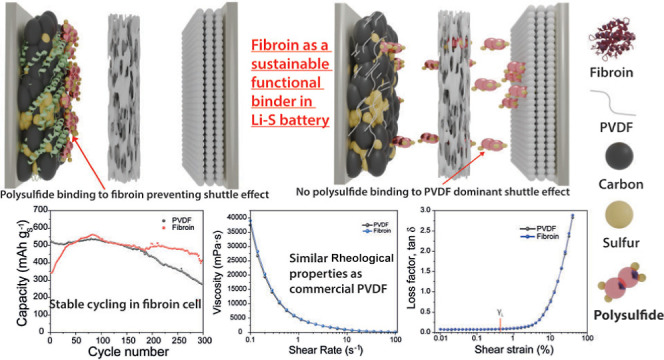

## Introduction

While lithium–sulfur (Li–S) batteries are known to offer a high theoretical energy density (2700 Wh kg^–1^) using low-cost and comparatively sustainable materials,^1^ several critical challenges must be overcome before this battery chemistry can be widely commercialized. The polysulfide shuttle effect is just one of the major issues that negatively affects Li–S battery capacity and cycling performance,^2^ along with the poor conductivity of sulfur and significant cathodic volume change that occurs during charge–discharge cycles and leads to positive electrode structural degradation. To address these challenges, researchers have focused on developing advanced composite sulfur positive electrodes^3,4^ and various support materials such as carbon nanotubes,^3^ carbon nanofibers,^4^ graphene,^5^ and catalytic additives like tellurium^6^ and covalent organic framework^7^ materials. However, these approaches introduce complications in the cell fabrication and can negatively impact the mass of the electrodes, making them less cost-effective and commercially viable.

Binders play a crucial role in the battery electrode structure, acting to hold active and conductive electrode particles together and ensuring that the composite electrode material adheres strongly to the current collector, which helps to maintain the battery’s structural integrity. Polyvinylidene fluoride (PVDF) is still the most used binder in several battery systems, including Li–S batteries, due to its excellent chemical, thermal, and mechanical stability and its resistance to degradation across a wide voltage range.^8^ However, the use of PVDF commonly necessitates the use of toxic, high-boiling-point, and expensive *N*-methyl-2-pyrrolidone (NMP). As it dries, PVDF also has a tendency to block carbon pores, hindering mass transport of Li^+^ in Li–S batteries.^9^ Importantly, PVDF is also harmful to the environment and extremely difficult to separate from other electrode materials, meaning it poses a significant barrier to the recycling of battery materials as they reach their end of life.^10^ There is therefore a critical need to find cost-effective and environmentally friendly alternatives to PVDF as a binder for a wide array of batteries to reduce their environmental impact. However, for Li–S batteries, it would also be ideal if any sustainable binders could help to overcome the drivers of cell degradation, such as polysulfide shuttling.

Biological polymers have been identified as an interesting class of materials that could offer an alternative to conventional battery binders due to their ready availability, nontoxic properties, and low environmental toxicity. Among the explored alternatives to PVDF, nonfluorinated, water-soluble polymeric materials such as poly­(acrylic acid), carboxymethyl cellulose, styrene–butadiene rubber, alginate, glycol chitosan, and various sugars (e.g., d-galactose, l-rhamnose, and l-arabinose), xanthan gum, and biobased polymers like poly­(3-hydroxybutyrate-*co*-4-hydroxybutyrate) and poly­(hydroxybutyrate-*co*-hydroxyvalerate) have been tested in Li-ion batteries.^11^ Specifically, in Li–S batteries, the use of Carbonyl-β-Cyclodextrin as a water-soluble binder in sulfurized polyacrylonitrile-based cells has been shown to offer stable performance over 50 cycles.^12^ Additionally, Na-alginate binders in Li–S electrodes have shown superior performance compared to PVDF-based electrodes, retaining 64.5% of capacity after 50 cycles, compared to just 31.2% retention in PVDF electrodes.^13^ Gel-forming materials such as guar gum,^14^ xanthan gum,^15^ and gum Arabic^16^ have also been applied as water-soluble binder materials in Li–S batteries. However, these materials show low cycle life due to the rampant polysulfide shuttle effect, as they have no significant affinity for polysulfides to impede their movement within the cell.

Some biological polymers have been shown to have robust interactions with molecules such as polysulfides, as they possess an abundance of intrinsically polar functional groups. Soy protein (SP), for example, has been demonstrated as an effective electrode additive to enhance polysulfide affinity, but it alone was shown to be ineffective as a standalone binder material. Hence, it needed to be combined with poly­(acrylic acid) to offer a synergistic effect that enhances overall binder performance in Li–S batteries.^55^ We previously demonstrated the versatility of the biological polymer fibroin as a multifunctional electrolyte additive in Li–S batteries,^17^ showing that its amino and carboxylic groups exhibit a strong affinity toward polysulfides and lithium ions, helping to prevent polysulfide shuttling and dendrite formation. However, a turbid electrolyte solution was obtained after dissolving fibroin, due to its low solubility in the Li–S battery electrolyte, which also increased electrolyte viscosity, hindering ionic conductivity.

Fibroin possesses a fibrous structure defined by a repetitive amino acid sequence. Within this structure, β-sheet nanocrystals act like nanoreinforcing rods, while the amorphous chains add toughness and provide many polar sites—this unique combination distinguishes it among natural polymers. This architecture effectively addresses the dual needs for strong adhesion to carbon and sulfur, alongside the flexibility required to accommodate the expansion and contraction of the cathode.^18^ Given these properties, fibroin can stand out as a promising candidate for use as a binder in Li–S cathodes.^19^ In this study, we investigate the potential of fibroin as a novel multifunctional binder in sulfur–carbon composite positive electrodes for Li–S batteries. Through analyses of fibroin’s binder properties using rheological, physical, and electrochemical methods, we demonstrate that fibroin can be effectively utilized as a drop-in replacement for PVDF, enabling the use of aqueous slurries yet offering comparable capacity, stability, and manufacturing performance in both coin and pouch cells. Importantly, we also show that the use of fibroin as a binder has the potential to revolutionize the recyclability of Li–S batteries, as by exploiting the denaturation of the protein when exposed to acidic conditions, all electrode components can be quickly and easily recovered for recycling or reuse.

## Experimental Section

### Materials

Sulfur, Cyrene (dihydrolevoglucosenone), poly­(vinylidene fluoride), and fibroin (∼100 kDa molecular weight) were purchased from Sigma-Aldrich and used as received. Timcal carbon C65 and lithium metal disks (15.5 mm diameter with 0.45 mm thickness) were purchased from PI-KEM Ltd. The electrolyte was prepared with 1, 3-dioxolane (DOL), 1, 2-dimethoxyethane (DME), lithium bis­(trifluoromethanesulfonyl)­imide (LiTFSI), and lithium nitrate (LiNO_3_) were provided by Sigma-Aldrich. AvCarb P50 carbon paper was purchased from the Fuel Cell Store.

### Electrode Preparation

For electrode fabrication, carbon and sulfur were first mixed with a mortar and pestle in a 77:23 ratio, which was then heated in a Teflon-lined autoclave at 155 °C for 12 h for melt infusion of sulfur inside the carbon pores.^20^ This sulfur–carbon composite obtained after heating and binder PVDF or fibroin was then mixed with Cyrene via ball milling (300 rpm for 1 h in Retsch PM 100) in a 90:10 weight ratio; the total solid content in the slurry was kept at 17% wt./v. The final composition in the electrodes was 70 wt % sulfur, 20 wt % carbon, and 10 wt % binder (PVDF or fibroin). The slurry was then drop coated on a carbon (AvCarb P50) current collector (14 mm disk), which was then dried on a hot plate at 50 °C for 6 h in ambient air. Before cell fabrication, the electrodes were dried in a vacuum oven at 40 °C for 12 h. Sulfur loading on the electrodes was between 3 and 3.5 mg/cm^2^.

### Coin Cell Fabrication

Two-electrode CR2032-type coin cells were constructed by stacking a lithium disk (diameter 15 mm), a Celgard 2400 separator (diameter 16 mm, 25 μm), and a sulfur–carbon positive electrode (diameter 14 mm). The electrolyte used in the cell contains 1 M LiTFSI, 0.8 M LiNO_3_, and a 1:1 v/v mixture of DOL/DME. The enclosure includes two 0.5 mm spacers and one 1.2 mm high/0.3 mm thick spring. The electrolyte-to-sulfur (E/S) ratio was maintained at 10 μL mg_S_^–1^ for all the cells.

### Pouch Cell Fabrication

For pouch cell fabrication, Li foil (Goodfellow, 0.12 mm thick and 14 × 25 mm dimensions), a positive electrode (14 × 25 mm) prepared by blade coating on a carbon-coated aluminum collector, and Celgard 2400 (25 μm, 16 × 35 mm) were stacked together inside an aluminum laminated pouch cell casing. The negative-to-positive capacity (N/P) ratio was measured 24 by considering the capacity of pure Li and sulfur used in the cell. The pouch cell was heat sealed at 200 °C after filling electrolyte (1 M LiTFSI, 0.8 M LiNO_3_, and a 1:1 v/v mixture of DOL/DME). The electrolyte ratio and positive electrode composition were kept the same as those used in coin cells.

### Electrochemical Measurements

Galvanostatic charge–discharge and cyclic voltammetry measurements (BSC-805 battery cycler) were used to show the batteries’ state of charge over time during the charging and discharging cycles. Electrochemical impedance spectroscopy (EIS) measurements were conducted at room temperature using a VSP Biologic multichannel potentiostat. Potentiostatic EIS measurements were carried out with an applied amplitude of 5 mV under open-circuit conditions. The frequency sweep ranged from 1 MHz to 50 mHz, progressing from higher to lower frequencies with 10 data points collected per decade for each EIS measurement. Following fabrication, the cells were allowed to rest for 2 h to ensure the electrode was thoroughly wet and stabilized before commencing measurements. The initial discharge of the cell is performed to bring it down to 1.8 V at a rate of C/20. This is followed by a complete cycle of full charging and discharging within the voltage range of 1.8–2.8 V, also at a rate of C/20. Subsequently, the cells are kept at open circuit voltage (OCV) for 45 min to ensure the establishment of a steady state before conducting EIS measurements.

### Rheology Analysis

The rheological properties were tested using an Anton Paar 702e rheometer, employing cone and plate geometry. The cone had a diameter of 49.96 mm and an angle of 1.023°. A constant gap of 1.07 mm and a temperature of 25.00 ± 0.01 °C were maintained throughout the measurements. The slurry viscosity was determined by varying the shear rate from 0.1 to 100 s^–1^. An amplitude sweep was conducted at an angular frequency of 10 rad s^–1^, spanning strain amplitudes from 0.1 to 100%, to establish the linear viscoelastic (LVE) regime. Additionally, a frequency sweep was performed over the range of 0.1–100 rad s^–1^. The positive electrode slurry was formulated to consist of 10% PVDF or fibroin, 20% carbon (C65 carbon), and 70% sulfur by weight. For rheological measurements, the composite was prepared by adding Cyrene in a ratio of 1 mg of composite to 0.02 mL of Cyrene, optimizing the slurry’s consistency and flow properties. Tensile tests of fibroin and PVDF films were assessed using an Instron 68SC-1 universal testing machine. Silk fibroin was dissolved in deionized water, and PVDF in NMP, with both solutions prepared at identical polymer concentrations; fibroin and PVDF solutions were dried in a Petri dish under ambient conditions to yield freestanding films with an areal mass loading of 14.5 mg cm^2^. Rectangular specimens (11 mm × 22 mm) were cut from the film centers, with thicknesses of 0.11 ± 0.01 mm (fibroin) and 0.09 ± 0.01 mm (PVDF) measured using a digital micrometer. Samples were mounted between pneumatic grips set to 2 bar and elongated to fracture at a cross-head speed of 5 mm min^1^ while engineering stress–strain data were recorded. Similarly, tensile tests were conducted for electrode sheets fabricated with PVDF and fibroin binder; the size of the sheets used was 25 × 45 mm with a thickness of 0.06 mm, using the same parameters as binder films.

### X-ray Computed Tomography

The Swagelok cell for in situ analysis was assembled using 1/8″ PFA Swagelok unions (model PFA-220–6 from Swagelok) with lithium foil (3.175 mm diameter) as the negative electrode. The Celgard-2400 (25 μm) served as the separator between the negative electrode and positive electrode, and a piece of glass fiber (Whatman GF/D, also 3.175 mm diameter) was used as the middle separator. A lab micro-CT instrument (ZEISS Xradia 620 Versa, Carl ZEISS Inc. Dublin, CA, USA) was used for X-ray tomography of the electrode structures. This instrument features a polychromatic microfocus source with a tungsten target, set at a tube voltage of 60 kV and equipped with a low-energy filter. An optical magnification of 20× was applied, and the CCD detector (2048 × 2048 px) was set to a binning of 2, achieving a pixel size of approximately 780 nm and a field-of-view around 750 μm. The tomographic data comprised 1601 radiographic projections at distinct angular intervals, which were then reconstructed into a series of tomographic slices forming a cylindrical volume using a cone-beam filtered back projection algorithm (XM Reconstructor, Carl ZEISS Inc., Dublin, CA, USA). The segmentation of images and analysis of volume fractions of electrode components were performed using Avizo 3D 2022.1 (Thermo Fisher Scientific in the UK).

### Optical Fluorescence Microscopy

Optical fluorescence microscopy analysis was performed to check the migration of polysulfide in the cell using the procedure demonstrated by Coke et al.^21^ The fluorophore 2-butyl-6-nitro-1*H*-benzo­[de]­isoquinoline-1,3­(2*H*)-dione (PS–Li_2_S_*x*_) was first synthesized through a procedure outlined in Figure S1a. 4-Nitro-1,8-napthalic anhydride (214 mg, 1 mM, Sigma-Aldrich, 95% purity) was first dissolved in 20 mL ethanol, and into this was added dropwise a mixed solution of butylamine (146.28 mg, 2 mM, Sigma-Aldrich, 99.5% purity) and triethylamine (200 μL, Fisher, 99.7% purity). This mixture was heated for 6 h under reflux conditions before purification via rotary evaporation and silica gel column chromatography using a dichloromethane (DCM, anhydrous, Sigma-Aldrich, ≥99.8% purity) and methanol (anhydrous, Sigma-Aldrich, 99.8% purity) (v/v, 15:1) eluent. 2-butyl-6-nitro-1*H*-benzo­[de]­isoquinoline-1,3­(2*H*)-dione (PS–Li_2_S_*x*_) was attained as the final product, as a brown solid (221.19 mg, 74.1% yield).

A cell was then built for operando fluorescence experiments using the EL-CELL ECC-Opto-Std optical cell in the side-by-side imaging procedure [as seen in Figure S1b]. The cell was constructed to a standard broadly seen in electrochemical studies across Li–S literature, with a 120 μm lithium foil negative electrode (Sigma-Aldrich) and a 70 wt % sulfur/20 wt % carbon composite positive electrode with 10 wt % PVDF or 10 wt % fibroin binder as electrodes. The electrodes were cut into semicircles of 14 mm diameter and placed over a glass fiber separator (EL-CELL) with an approximately 3 mm gap between them to be imaged. 70 μL of electrolyte (1 M LiTFSI and 0.8 M LiNO_3_ in 1:1 (v/v) DOL/DME) with 20 μM of the fluorescent dye PS-Li_2_S_*x*_ was added via pipet to the separator. The exposed region of glass fiber between the two electrodes was focused on to represent the electrolyte—with the fluorescence intensity emitted during operando cycling proportional to the polysulfide concentration.

A Gamry Interface100 potentiostat was employed for charge–discharge cycling of the cell, using a current density of 0.7 mA cm^–2^ with respect to the lithium metal negative electrode. A ZEISS Axio Zoom V16 zoom microscope was employed for the fluorescence imaging during these studies, alongside a ZEISS illuminator HXP 200C fluorescence light source. Imaging was conducted with the EXview HAD CCD II camera (ZEISS with Cam 506 color) and the ZEISS Filter Set 38 HE was used to filer excitation and emission light wavelengths. Image analysis was conducted with the ZEISS ZEN 3.6 pro software and within MATLAB using custom scripts.

### Scanning Electron Microscope Analysis

Scanning electron microscopy (SEM) was performed using a Zeiss EVO MA10 (Carl ZEISS AG, Germany) microscope. For chemical identification, energy dispersive X-ray spectroscopy (EDX) was applied with an AztecONE detector (Oxford Instruments, Oxford, UK).

### Examining Fibroin and PVDF Solubility in Electrolyte Solvents

A qualitative solubility analysis of PVDF and fibroin in a DOL/DME solvent mixture was performed by adding 10 mg of either PVDF or fibroin to 1 mL of the DOL/DME (1:1 v/v) solution. The mixture was stirred for 24 h inside a glovebox. Both fibroin and PVDF demonstrated minimal solubility in the DOL/DME mixture; however, fibroin exhibited slightly higher turbidity, indicating a little higher solubility than PVDF (Figure S2). Previously, fibroin was demonstrated as an electrolyte additive in Li–S cells, in which the maximum concentration was 0.8% wt./v prepared by sonication; the fibroin solution at this concentration was turbid, showing the limit of fibroin solubility.^17^

## Results and Discussion

The amino group’s nitrogen atom in fibroin has been shown to have a notably strong affinity for binding to polysulfides, effectively mitigating the shuttle effect observed during electrochemical reactions.^17^ To investigate the properties of fibroin as a drop-in replacement binder, a comparative evaluation of electrodes fabricated with a fibroin binder was performed against PVDF, ensuring an equivalent composition across both cells. The outcomes of electrochemical testing, as outlined in Figure 1, reveal that during the initial charge–discharge cycle at a current density of C/20 (Figure 1a), the fibroin-containing cells and PVDF-containing cells achieved nearly equal capacities of 987 mAh g_(S)_^–1^ and 958 mAh g_(S)_^–1^, respectively. CV profiles indicate that both cells exhibit similar kinetic behavior for the conversion of sulfur to polysulfides (0 to 700 mAh g_(S)_^–1^), although slightly slower kinetics for the conversion of Li_2_S_2_ to Li_2_S (700 to 961 mAh g_(S)_^–1^) are seen for the fibroin-containing cell. Nonetheless, such similarity in capacities and voltage profiles underscores the fact that fibroin, a sustainable and bioderived material, can act as an effective binder for Li–S batteries.

**Figure 1:**
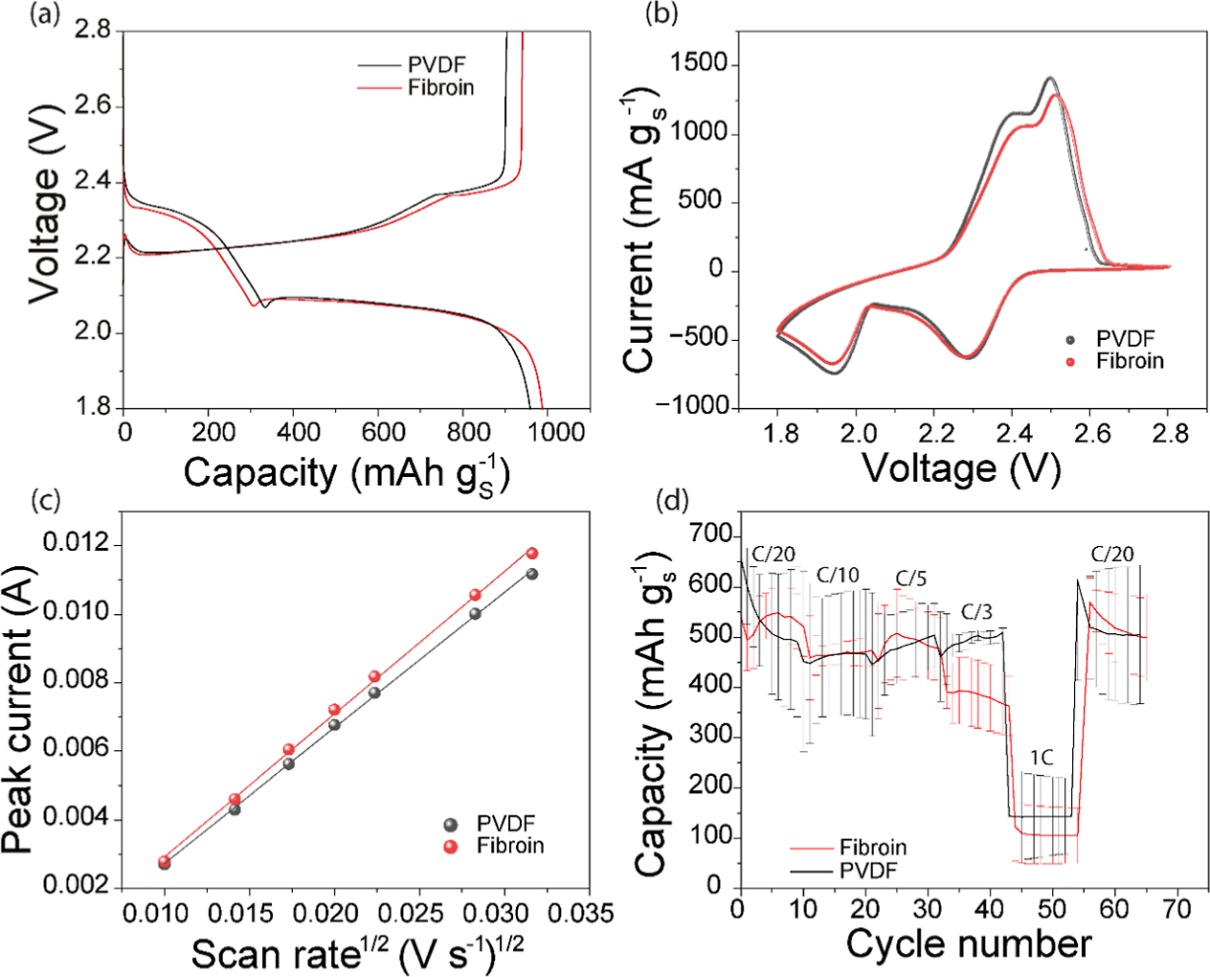
Comparison of the electrochemical behavior of fibroin with PVDF. (a) First charge–discharge cycle measured at C/20 current density; (b) cyclic voltammogram recorded at a scan rate of 0.2 mV s^–1^; (c) Shows the linear fit of CV peak currents at different scan rates; (d) shows the rate behavior of fibroin and PVDF.

Cyclic voltammetry (CV, Figure 1b) analyses of cells incorporating fibroin and PVDF, conducted at a scan rate of 0.2 mV s^–1^, exhibit consistent electrochemical behavior, particularly in terms of peak numbers and positions across varying scan rates (refer to Figure S3 for CV at different scan rates). During the discharge, a peak at 2.3 V is indicative of high-order polysulfide formation, while a peak at approximately 2 V signals the generation of solid Li_2_S_2_/Li_2_S.^22^ Conversely, during charging, peaks at 2.4 and 2.5 V are representative of the formation of Li_2_S_2_ and Li_2_S_6_, respectively.^23^ These CV results further reinforce the assertion that fibroin maintains the intrinsic electrochemical integrity of the Li–S cell, thereby highlighting its potential viability as a binder material. The Randles-Sevcik eq 1 describes the effect of scan rate on peak current in cyclic voltammetry, and it also relates peak current with scan rate

1 $$i_{p}=2.69\times 10^{5}n^{3/2}AC{\left(Dv\right)}^{1/2}$$

where *i*_p_ is the peak current density in Amps, *n* is the number of electrons transferred in the redox event, A is the electrode area in cm^2^, *D* is the diffusion coefficient cm^2^ s^–1^, C is the concentration in mol cm^–3^, v is the scan rate in *v* s^–1^.^24^ The peak current (*i*_p_) vs scan rate (v) plot for fibroin and PVDF is shown in Figure 1c; the slope of the line gives information about the diffusion properties of the electrode.^25^ The slope measured for PVDF is 0.396, while for fibroin the slope measured is 0.417. While the extraction of definitive diffusion coefficients using Randles-Sevcik analysis is often inexact for complex electrodes, the higher slope for the fibroin electrode may indicate improved ion-diffusion properties. Fibroin films have previously been reported to exhibit Li^+^ conductivity (5.8 mS cm^–1^), which can explain improved Li^+^ diffusion in fibroin electrodes.^26^ Rate performance analysis, shown in Figure 1d, indicates comparable trends for both fibroin and PVDF at varied current densities, including C/20, C/10, C/5, and C/1. Only at C/3 in the data shown is there a discernible difference between the cells utilizing fibroin compared to those with PVDF, but while this may suggest a weaker intrinsic performance with fibroin, it is more likely due to the fact that the fibroin and PVDF electrodes have been prepared with a procedure optimized for the traditional PVDF binder. Further optimization of electrode porosity and conductivity would likely overcome this limitation, as suggested by data highlighting the effectiveness of fibroin as a binder (below).

To assess the impact of fibroin on the cycling stability of the Li–S cell, charge–discharge at a current density of C/5 was carried out for 300 cycles. The stability data of fibroin is compared with durability in Figures 2a and S4, showing longer and more stable cycling than PVDF. In fibroin cells, the capacity increases up to ∼60 cycles, possibly due to activation or increased wetting by the electrolyte,^27^ reaching a maximum and stabilizing afterward. We conducted contact angle measurements to evaluate electrode wetting by the electrolyte (Figure S5). Upon dropping the electrolyte on both fibroin and PVDF binder electrodes, it spread rapidly and was fully absorbed in less than 6 s, demonstrating high wettability for both types of electrodes. This suggests that electrode wettability does not contribute to the slow activation of sulfur at elevated charging rates in fibroin cells. In contrast to fibroin cells, PVDF binder shows stable performance only up to 100 cycles cells and degrades constantly afterward. Furthermore, electrodes fabricated with fibroin show slightly improved Coulombic efficiency with an average Coulombic efficiency of 94.15% compared to the 93.83% average Coulombic efficiency measured for PVDF cell (Figure 2b).

**Figure 2:**
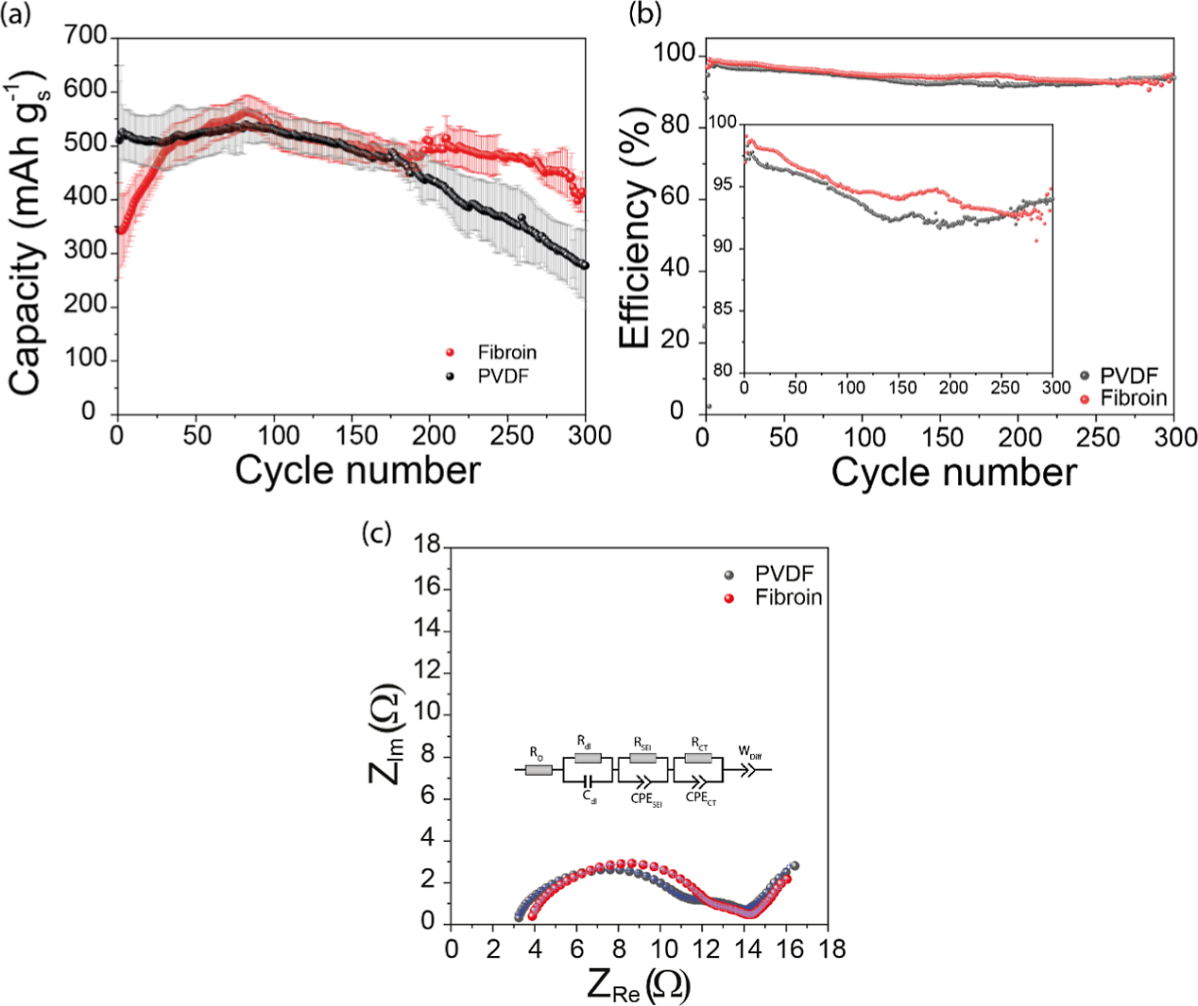
(a) Stability data of fibroin and PVDF binder cells at C/5; (b) shows cycling efficiency; (c) Nyquist plot fitted with the circuit shown inside the figure.

Electrochemical Impedance Spectroscopy (EIS) measurements were also conducted to determine the internal resistance characteristics of the Li–S cells containing fibroin and PVDF. The impedance spectra (Nyquist plots) of the cell with PVDF and fibroin, depicted in Figure 2c, show a large depressed semicircle in the high-mid frequency region, a small semicircle in the midlow frequency region, and a diffusion line in the low frequency region. To isolate the resistance parameters of the two cells, Nyquist plots were fitted with equivalent circuits, shown in the inset of Figure 2c, where *R*_O_ represents equivalent series resistance (mostly contributed by electrolyte resistance); *R*_dl_ and *C*_dl_ represent resistance and capacitance of the electrical double layer; *R*_SEI_ and CPE_SEI_ represent the solid electrolyte interface, *R*_CT_ and CPE_CT_ represent charge-transfer resistance at the positive electrode; and *R*_Diff_ indicates Li-ion diffusion resistance in the sulfur electrode.^28^ The values of the equivalent circuit parameters of the two cells are given in Table S1. The first semicircle can be attributed to the double-layer charging of the electrochemical interface with components *R*_dl_ pertaining to resistance associated with double-layer relaxation, having a value of 2.71 Ω for the PVDF cell and 1.38 Ω for the fibroin cell. The second semicircle represents the solid electrolyte interface of the Li-negative electrode, where *R*_SEI_ is the charge transfer resistance for the Li-ion migration through SEI and plating/stripping. The fibroin cell shows higher *R*_SEI_, 6.87 Ω, compared to 4.35 Ω for the PVDF cell; the higher value in fibroin is possibly caused by the variation in the SEI of the negative electrode after the formation cycle. The third semicircle pertains to the electrochemical reactions at the positive electrode, where fibroin and PVDF are present in the respective electrodes and can give information about their effect in the sulfur electrochemistry. The *R*_CT_ with a value of 2.24 Ω is 43% lower in the fibroin cell compared to 3.92 Ω in the PVDF cell; a low value of *R*_CT_ indicates faster electrode kinetics in the fibroin cell, which is also indicated by the CV measurements (Figure 1b,c). The final component of the impedance spectra is the diffusion line in the low-frequency region represented by the constant phase element (CPE) *W*_diff_. This signifies the diffusion of Li-ion in the porous electrode (positive electrode). Warburg impedance (diffusion impedance) is related to the CPE by

2 $$\sigma =\frac{1}{\sqrt{2}Q_{0}}$$

where *Q*_O_ is the capacitance of CPE, which in turn is related to the Li-ion diffusion as $D_{Li}\alpha \frac{1}{\sigma 2}$ (3), meaning a higher Warburg impedance (σ) indicates a slower Li-ion diffusion (*D*_Li_) in the electrode.^29^

The Warburg impedance calculated for the fibroin cell is 1.006, while a higher value of 1.268 is obtained for the electrode fabricated with PVDF binder, suggesting improved Li-ion transfer in the fibroin electrode, supporting the Randles-Sevcik analysis. Molecular dynamics simulation^17^ studies have shown high coordination of Li-ions with fibroin, indicating Li-ions are present in close vicinity of the sulfur/carbon interface bounded by fibroin, which helps explain the impedance observations for fibroin binder. The performance of fibroin as a binder is compared in Table S2 with various organic binder materials that have been used in Li–S batteries. Most of these studies use sulfur loadings ranging from 35–60 wt % and surface loadings of 0.5 to 1.5 mg cm^–2^. The table demonstrates that the high-sulfur-loading, commercially relevant cells fabricated with fibroin in this study outperform other organic binders in terms of cycling stability and capacity retention. This highlights fibroin’s exceptional potential as an effective binder material for Li–S batteries, further reinforcing its suitability for high-performance applications in this field.

The electrochemical testing described above revealed that fibroin acts as a strong and stable binder for Li–S battery positive electrodes. To elucidate the underlying mechanisms of fibroin driving these advanced binder properties, physical, optical, and mechanical characterization tools were applied. Illustrated in Figure 3, SEM images show microstructural characteristics of fibroin-based electrodes (Figure 3a) and PVDF electrodes (Figure 3b), highlighting similar morphologies characterized by small-sized carbon particles with open porosities and sulfur aggregations dispersed across the examined regions.

**Figure 3:**
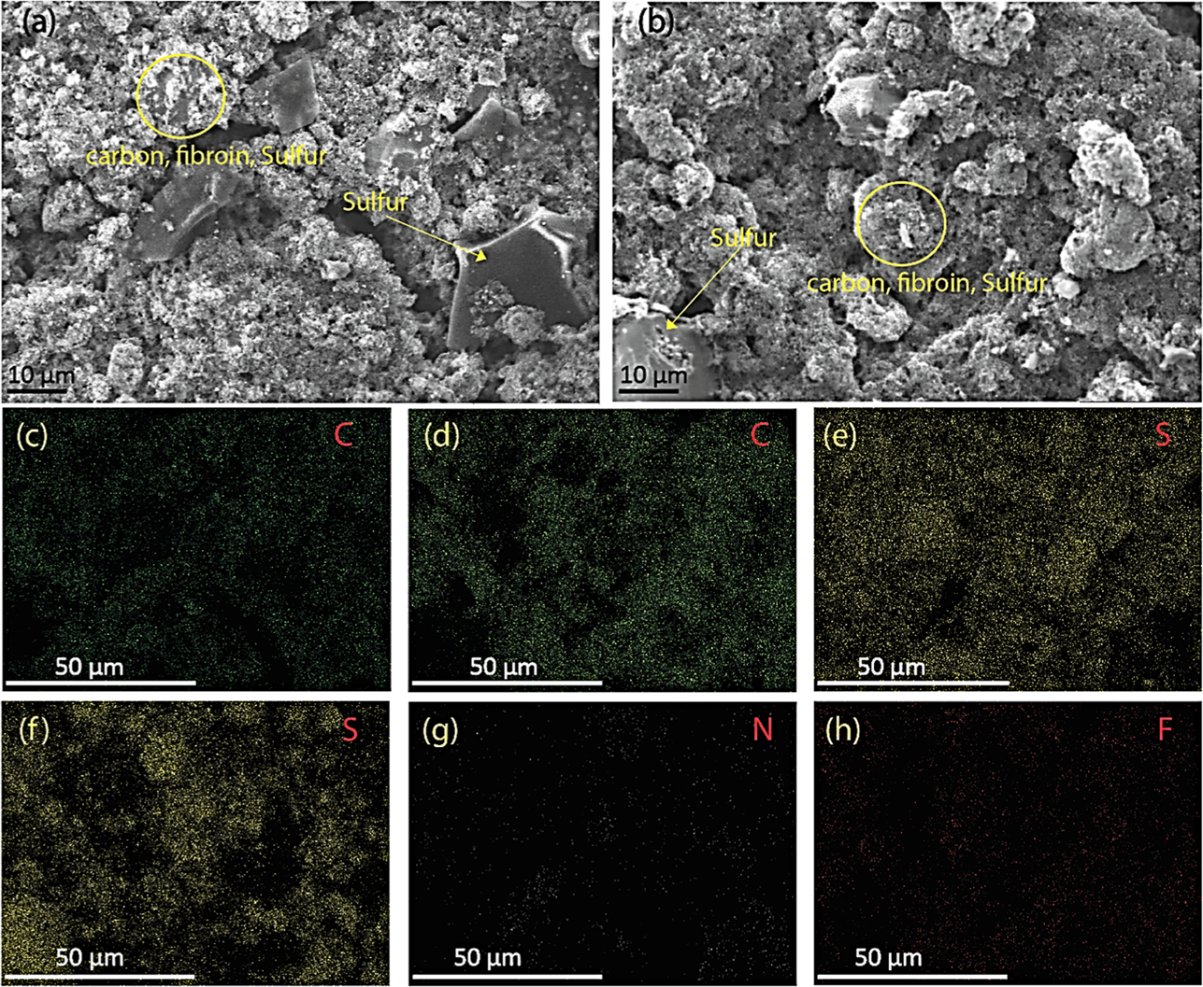
(a) Scanning electron microscope image of electrode fabricated with fibroin binder; (b) SEM of electrode containing PVDF binder; (c,e,g) Element maps of carbon, sulfur, and nitrogen present in the fibroin electrode, respectively; (d,f,h) Show the element distribution of carbon, sulfur, and fluorine present in electrodes containing PVDF binder, respectively.

Energy Dispersive X-ray Spectrometry (EDX) maps for fibroin (Figure 3c,e,g, representing carbon, sulfur, and nitrogen, respectively) and for the PVDF electrode (Figure 3d,f,h, correspondingly) revealed a homogeneous distribution of these elements within the scanned areas. Nitrogen (Figure 3g), although weak in intensity (light elements have weak EDX signal),^30^ is present everywhere in the image, confirming good dispersion of fibroin throughout the electrode. Elemental analysis also shows the presence of carbon, sulfur, oxygen, nitrogen, and fluorine (Figure S6). In PVDF electrodes, the fluorine and oxygen content are 2.5 and 3.7 wt %, respectively. In contrast, the fibroin electrode has nitrogen and oxygen contents of 2.3 and 3.3 wt %, respectively. Notably, nitrogen and fluorine are present in similar amounts; however, the inclusion of protein enhances the cycle life of the electrode.

SEM can only provide information about the surface structure and composition. Therefore, to obtain insight into the bulk electrode composition, X-ray computed tomography (X-ray CT) was employed to investigate the dispersion of electrode constituents within the fibroin and PVDF-binder-based electrodes. Virtual slices extracted from micro-CT scans of cells fabricated with positive electrodes containing fibroin and PVDF binder are illustrated in Figure 4; in both fibroin and PVDF cells, sulfur (Figure 4c,g) appears as light regions, whereas carbon and the binder (Figure 4b,f) manifest as darker regions (binder and carbon are difficult to differentiate with X-ray CT due to their similar X-ray absorption cross sections).

**Figure 4:**
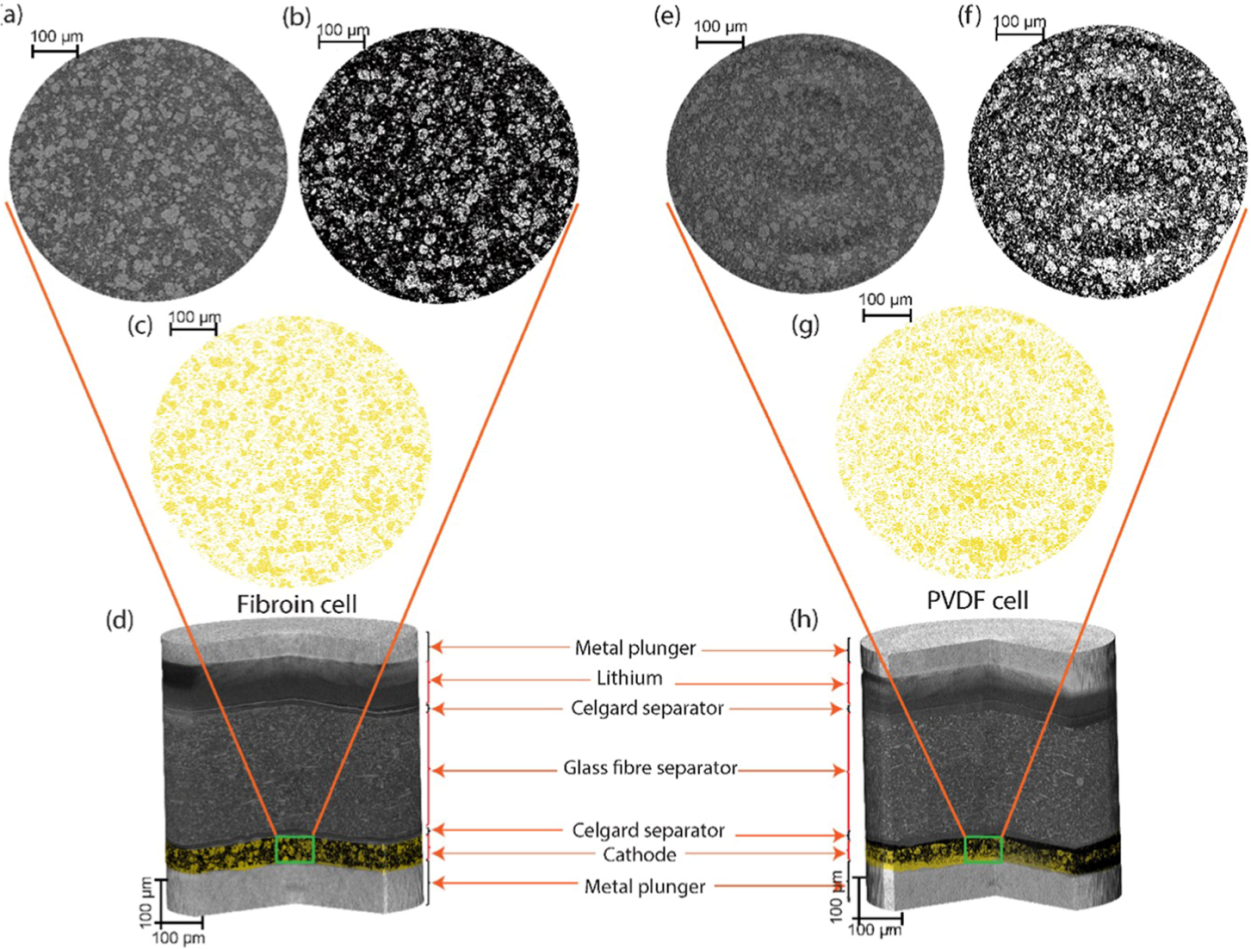
X-ray computed tomography. (a,e) Virtual slices of tomographic volume of fibroin cell and PVDF cell, respectively; (b,f) are the 3D rendering of carbon binder phase in fibroin and PVDF cell, respectively; (c,g) illustrate 3D rendering of sulfur phases in fibroin and PVDF cell, respectively; (d,h) volume rendering of the fibroin and PVDF cell demonstrating the constituents of the full cell.

Examining a virtual slice of the positive electrodes allows the delineation of the materials within the positive electrodes, showing that in both the fibroin and PVDF cells, carbon (Figure 4b,f, respectively) and the sulfur-binder domain (Figure 4c,g) showed similar characteristics with uniformly distributed carbon and sulfur in the electrode. Uniform and even distribution of the electrode’s constituents is crucial for maximizing material utilization, ensuring rapid ion diffusion, and maintaining mechanical strength. These factors are particularly significant for fibroin electrodes, demonstrating an ability to evenly disperse electrode components—a key requirement for effective binder materials.

Further X-ray CT slices of representative cross sections from the fibroin and PVDF cells, measured in both a pristine state and discharged state (discharged to 1.8 V at C/10 rate to 0% state-of-charge; SoC), are shown in Figure 5. Comparative analysis highlights significant morphological changes. In the pristine state (100% SoC, Figure 5a), the fibroin cell exhibited a well-defined carbon and sulfur structure within the positive electrode, which became unresolvable postdischarge as sulfur converts to lithium sulfide (0% SoC, Li_2_S, Figure 5b). Li_2_S is not distinguished at 0% SoC due to similar X-ray attenuation of Li_2_S and carbon. Once elemental sulfur is fully converted to Li_2_S, its linear mass-attenuation coefficient at the X-ray energy used becomes almost identical to that of the amorphous carbon matrix.^31^ This transformation is accompanied by a decrease in electrode thickness and a noticeable deformation in the lithium metal negative electrode, suggesting lithium migration toward the positive electrode during discharge. In Figure 5a, the thickness of the positive electrode in the pristine state is 55.3–56.4 μm which changed slightly after discharge to 48.2–52.9 μm and increased from 53.2 to 57.8 μm at a different location (Figure S7) due to expansion, also indicating nonuniform cathode expansion. In the PVDF cell (Figure 5c,d), the pristine state also shows a distinct structure in the positive electrode, which shrinks after the first discharge, decreasing from 45.4–46.2 μm to 26.4–29.1 μm.

**Figure 5:**
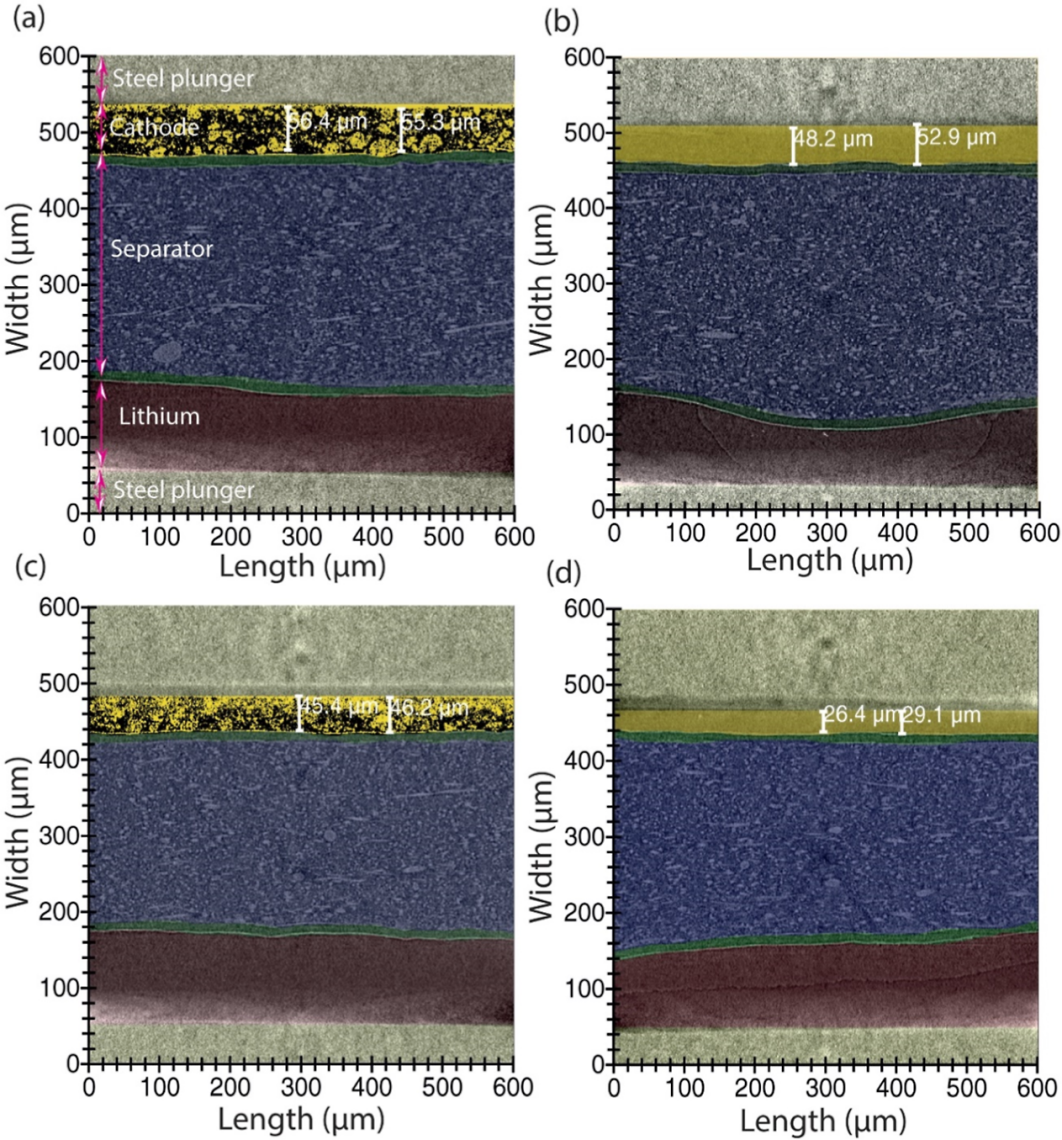
Cross-sectional slices of the tomograms. (a) Fibroin cell in pristine state; (b) Fibroin cell after discharge at C/10; (c) PVDF cell in pristine state; and (d) PVDF cell after discharge at C/10.

Interestingly, here the negative electrode did not show a significant change in thickness as observed in the PVDF cell (ranging from 103.6–124.5 μm when pristine and dropping to 102.1–120.2 μm after discharge, vs. 101.6–112.4 μm to 78.6–113.1 μm for the fibroin cell, see Figure S8a–d). Furthermore, the volume calculated for the pristine fibroin positive electrode was 0.0106 mm^3^ which increased to 0.0148 mm^3^ on discharge; the increase in thickness is expected as the density of Li_2_S (1.66 g cm^–3^) formed at the end of discharge is lower than that of sulfur (2.09 g cm^–3^) present in the pristine state. Interestingly, the PVDF positive electrode showed a decrease in volume after discharge from 0.0141 mm^3^ in the pristine state to 0.0097 mm^3^. The observed variation in the thickness and volume may be explained by differences in the sulfur loss; fibroin in the positive electrode binds polysulfides, preventing their movement to the negative electrode; therefore, the volume increased as it retained almost all of its active material, and for similar reasons, the thickness of lithium shrinks due to stripping and its migration to the positive electrode to form Li_2_S. However, in the PVDF cell, polysulfide shuttling is particularly active due to the absence of polysulfide binding forces, leading to their diffusion to the negative electrode, where they get oxidized and condensed.^32^ Further, the volume of Li in the pristine PVDF cell was 0.0464 mm^3^ which after discharge reduced to 0.0425 mm^3^, an 8.4% volume decrease. In a fibroin cell, pristine Li is 0.0420 mm^3^, reducing to 0.0362 mm^3^ after discharge, showing a 13.8% volume decrease. A low volume change in PVDF indicates high accumulation of Li_2_S/Li_2_S_2_ in Li of the PVDF cell. Hence, the thickness of lithium does not show appreciable change, whereas the positive electrode shows a significant reduction in thickness and volume, attributed to the loss of active material due to the shuttle effect. To confirm the X-ray CT observations in PVDF cells, these measurements were repeated. In this repeat cell, the thickness of the negative electrode increased from 90.4 to 101.7 μm and 94.1 to 98.6 μm at the edges and decreased slightly from 87.4 to 73.5 μm (Figure S8e,f) indicating polysulfide migration to anode and its reduction leading to an increase in negative electrode thickness. The thickness of the positive electrode also decreased along with volume decrease from 0.013 mm^3^ to 0.011 mm^3^, similar to the observation in Figure 5c,d, validating the results obtained in the PVDF binder cell (see Figure S9). The XCT results clearly show the prevalence of shuttle in PVDF cells and the polysulfide binding ability of fibroin preventing loss of active material and lithium negative electrode corrosion.

To further verify the efficacy of the fibroin binder in capturing polysulfides, optical fluorescence microscopy (OFM), as previously described in the work by Coke et al.,^21^ was used. In this method, a highly selective fluorophore is added into the Li–S electrolyte, producing a fluorescence response that is sensitive to the presence of lithium polysulfides.^21^ Within the linear range of the fluorophore (0.1 μM–100 μM) the fluorescence intensity increases linearly with polysulfide concentration, and so quantitative assessments of concentration can be made using calibration curves. At concentrations outside of this, a fluorescence quenching effect is observed, where increasing polysulfide concentration causes a qualitative linear decrease in fluorescence intensity, allowing qualitative assessment. The properties of the dye in Li–S cells have been previously established, including the excitation and emission wavelengths of λ_ex_ = 430 nm and λ_em_ = 535 nm, the trends in its fluorescence response with changing polysulfide concentration, its high degree of selectivity for lithium polysulfides, and the lack of impact on Li–S performance when added to the electrolyte of a standard Li–S cell.^21^

To assess the impact of the fibroin binder on polysulfide trapping using OFM, it was first necessary to establish that there were no detrimental interactions between the fluorescent dye (PS–Li_2_S_*x*_) and the fibroin. To do this, Li–S coin cells containing commercial positive electrodes (NEI Nanomyte BE-70, 70% sulfur, 20% carbon, 10% PVDF alongside a 0.8 wt % fibroin electrolyte additive, as developed in our previous work,^17^ were prepared both with and without the addition of 20 μM PS-Li_2_S_*x*_ into the electrolyte. As seen in Figure S10, there was no substantial deviation in either charge/discharge profile or capacity retention upon the addition of the fluorescent tag PS-Li_2_S_*x*_, with the slight differences observed within the expected natural variance of the commercial positive electrodes.^21^ PS-Li_2_S_*x*_ has also previously been shown to be inert to the other key components of a standard Li–S cell employed here (lithium metal negative electrode, 70% sulfur/20% carbon composite positive electrode, and electrolyte of 1 M LiTFSI, 0.8 M LiNO_3_, in a 1:1 v/v mixture of DOL/DME).^21^ Hence, the dye can be integrated into cells containing fibroin to prove to be a reliable indicator of electrolyte polysulfide concentration.

To test the polysulfide trapping ability of the fibroin binder, Li–S cells were created within a specialized operando optical microscopy cell employing a side-by-side cell imaging procedure as outlined in the experimental section.^33,34^ One cell was taken as a baseline for comparison, employing a standard electrolyte and using the PVDF binder, while the other cells included the fibroin binder. The cells were cycled at a current density of 0.7 mA cm^–2^ with respect to the geometric area of lithium to prevent the excess dendrite formation, noted to occur above 1 mA cm^–2^.^35^ Fluorescent light at the activation wavelength of 430 nm was shone at the electrolyte gap while images were taken every minute—with the resulting fluorescence intensity detected at each pixel indicative of the localized polysulfide concentration, according to the qualitative concentration quenching effect previously established, where increases in fluorescence intensity arise from decreases in polysulfide concentration.^21^ The results from these experiments, including the cycling data, fluorescent images taken at key mechanistic points, and the normalized polysulfide concentrations averaged for the negative electrode, positive electrode, and across the entire electrolyte, are given in Figure S11 for the first cycle and Figure 6 for the third.

**Figure 6:**
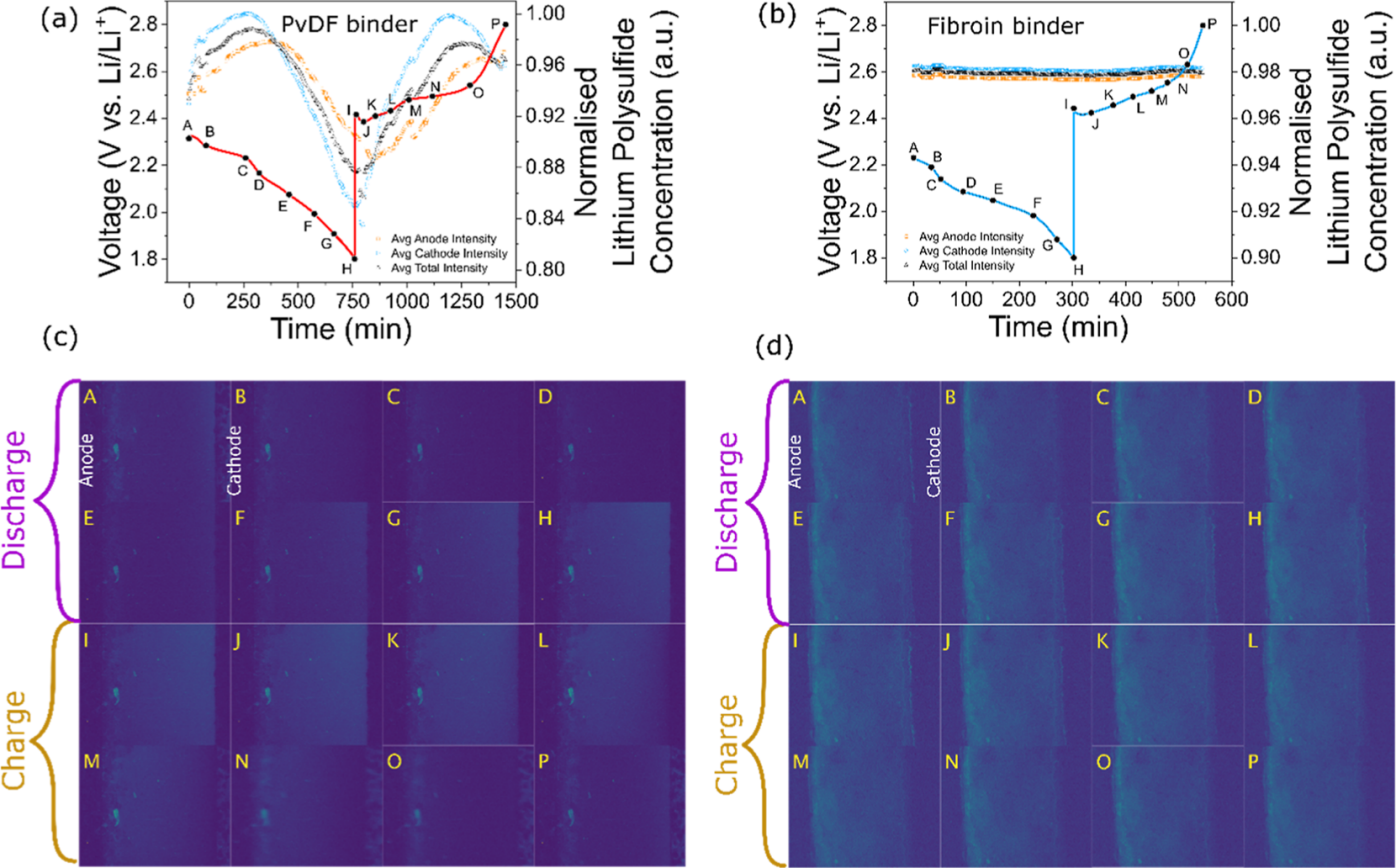
Cycling data with normalized polysulfide concentration (a,b) and optical fluorescence imaging (c,d) taken during the third cycle of an operando study of the electrolyte of a Li–S cell, with 20 μM polysulfide-sensitive PS-Li_2_S_*x*_ fluorescent dye. The cells utilized a PVDF binder and standard electrolyte (a,c) and a fibroin binder and standard electrolyte (b,d).

In the control Li–S cell, employing a PVDF binder with the standard electrolyte, the expected trends in polysulfide concentration across the electrolyte, as reported previously, were observed (Figures S11a,b and 6a,c).^21,36^ First, an initial decline in total average polysulfide concentration during the first discharge stage, as polysulfides in the electrolyte (created during S_8_ dissolution from self-discharge) are removed through Li_2_S_2_/Li_2_S deposition.^37,38^ The subsequent charge step sees a continual increase in total polysulfide concentration from Li_2_S_2_/Li_2_S dissolution, until the second charge plateau, at which point it declines because of the onset of S_8_ deposition. The occurrence of the polysulfide shuttle effect can also be seen in the fluorescence images (Figure S11b.K–N) as a sweeping darkness from the positive electrode to the negative electrode, which represents the mass diffusion of active material toward the highly reactive lithium metal negative electrode. The following cycles show largely the same trends, though begin with the sulfur predominantly deposited as S_8_ rather than solubilized through self-discharge and thus show an initial increase in polysulfide concentration at the first discharge plateau from the electrochemically induced S_8_ dissolution (Figure 6a,c). Crucially, between cycles, while the same general trends in polysulfide evolution are observed as described above, the fluorescence imaging depicts a decrease in the magnitude of the polysulfide concentration, corresponding to the loss of the sulfur active material as polysulfides in the electrolyte.

A polysulfide trapping effect is observed immediately upon the incorporation of a fibroin binder to the positive electrode of an Li–S cell (Figure S11b,d).^17^ In the fibroin cell, the lowest normalized polysulfide concentration (highest degree of fluorescence) seen across all cycles is observed when in the pristine state, before discharge induces any S_8_ dissolution (Figure S11d.A) indicating that the initial self-discharge to Li_2_S_8_ that was observed in the PVDF cell (Figure S11c.A) was being prevented by the polysulfide-trapping fibroin. Only as the first discharge plateau begins and drives S_8_ dissolution (Figure S11c.C), does the fibroin cell show any substantial increase in normalized dissolved polysulfide concentration (a decline in fluorescence) to near its highest point across all cycles. However, beyond this initial dissolution, minimal change is seen in the polysulfide concentration, appearing almost entirely constant throughout the rest of the first cycle (Figure 6b,d). This is in stark contrast to the PVDF cell, which sees large fluctuations during the first cycle owing to the continual dissolution of solid S_8_ to polysulfides during discharge and Li_2_S_2_/Li_2_S during charge (all of which are able to diffuse into the electrolyte) followed by their subsequent deposition back to solids (Figure 6a,c). This is a clear indication of a reduction in polysulfide concentration escaping the positive electrode and thus a reduction of the polysulfide shuttle phenomenon, arising from the implementation of the fibroin polysulfide trapping agent.

This effect remains apparent for all of the later cycles. During the third cycle, the concentration of polysulfides within the standard PVDF cell fluctuates between 16.59% of its total normalized polysulfide concentration (Figure 6a,c), whereas the cells with fibroin binder fluctuate within 0.26% of their total normalized polysulfide concentration (Figure 6b,d). While direct comparisons cannot be made between the two cells, owing to the qualitative nature of the technique at high concentrations, the cells were sufficiently consistent in sulfur mass and electrolyte composition that this drastic suppression in the variance of polysulfide concentration during the operando cycling is notable. It represents a fibroin-induced inhibition of polysulfide escape into the electrolyte during the cycling—and thus of successful polysulfide trapping and shuttle mitigation.

Further, a distinctive feature of the spatial fluorescence data for Li–S cells is where the spatially resolved polysulfide concentrations at each electrode crossover. At the end of the first discharge plateau, the observed polysulfide concentration near the negative electrode rises above that near the positive electrode, while the inverse is seen at the start of the first charge plateau: the polysulfide concentration near the positive electrode rises above that near the negative electrode. This is representative of the polysulfide shuttle effect, where during discharge the concentration at the negative electrode rises from polysulfides diffusing from the positive electrode, while at the positive electrode, the concentration decreases from Li_2_S_2_/Li_2_S deposition. These polysulfides react with the negative electrode to degrade its SEI and electrolyte, contributing to cell death. Notably, in the cell employing the fibroin binder, these crossover points cannot be seen to occur. The concentration of polysulfides near the negative electrode does not exceed that of the positive electrode at any point, again suggesting a negation of the polysulfide shuttle effect from the use of the fibroin binder. The high polysulfide binding ability of the fibroin, as observed in the fluorescence test, stems from the binding of polysulfide to the functional groups present in the amino acids of the fibroin. Fibroin primarily consists of alanine (30.3%), glycine (45.9%), serine (12.1%), while the remaining 11.7% comprises aspartate, asparagine, glutamic acid, glutamine, valine, leucine, arginine, tyrosine, proline, threonine, phenylalanine, histidine, lysine, and cysteine.^39^ Polar amino acids such as serine, tyrosine, threonine, cysteine, asparagine, and glutamine, as well as positively charged arginine, lysine, and histidine present in fibroin have been shown to possess a strong affinity toward polysulfides.^39^ Moreover, molecular dynamic simulation studies on fibroin showed that soluble long-chain polysulfide showed deep impregnation of fibroin at the center, meaning the soluble higher-order polysulfide molecules are trapped within the fibroin structure to prevent them from moving to the anode. Thus, fibroin possesses a great ability to immobilize polysulfides, alleviating the polysulfide shuttle effect. Also, the impregnation and coordination number of Li^+^ in fibroin decrease on interaction of fibroin with polysulfide, whereby it can release additional Li^+^ in the electrolyte upon polysulfide uptake, improving mass transport of Li^+^.^17^

After the utility of fibroin as a binder in Li–S positive electrodes is ascertained, it is necessary to understand how the use of fibroin would impact the manufacturability of Li–S cells. Hence, the coating quality of slurries made with fibroin was determined by performing a rheological analysis. The coating efficacy of electrode slurries is intricately linked to the interactions among their components, rendering rheology a pivotal determinant of their coating characteristics.^40^ The viscosity versus shear rate profiles for both fibroin- and PVDF-based slurries are depicted in Figure 7a, demonstrating a shear-thinning behavior common to both, where viscosity diminishes with increasing shear rate.^41^ These measurements, spanning a shear rate range of 0.1 to 100 s^–1^—pertinent to the operational parameters of the slot dye coating apparatus—reveal that at a low shear rate of 0.1 s^–1^, the viscosities for PVDF and fibroin slurries stand at 37.5 and 39.0 Pa·s, respectively, and at a higher shear rate of 100 s^–1^, they narrow to 0.16 for PVDF and 0.17 for fibroin. Such equivalent viscosity metrics underscore the similar gelation characteristics and stability of fibroin-based slurries to those constituted with the widely adopted PVDF binder.^42^

**Figure 7:**
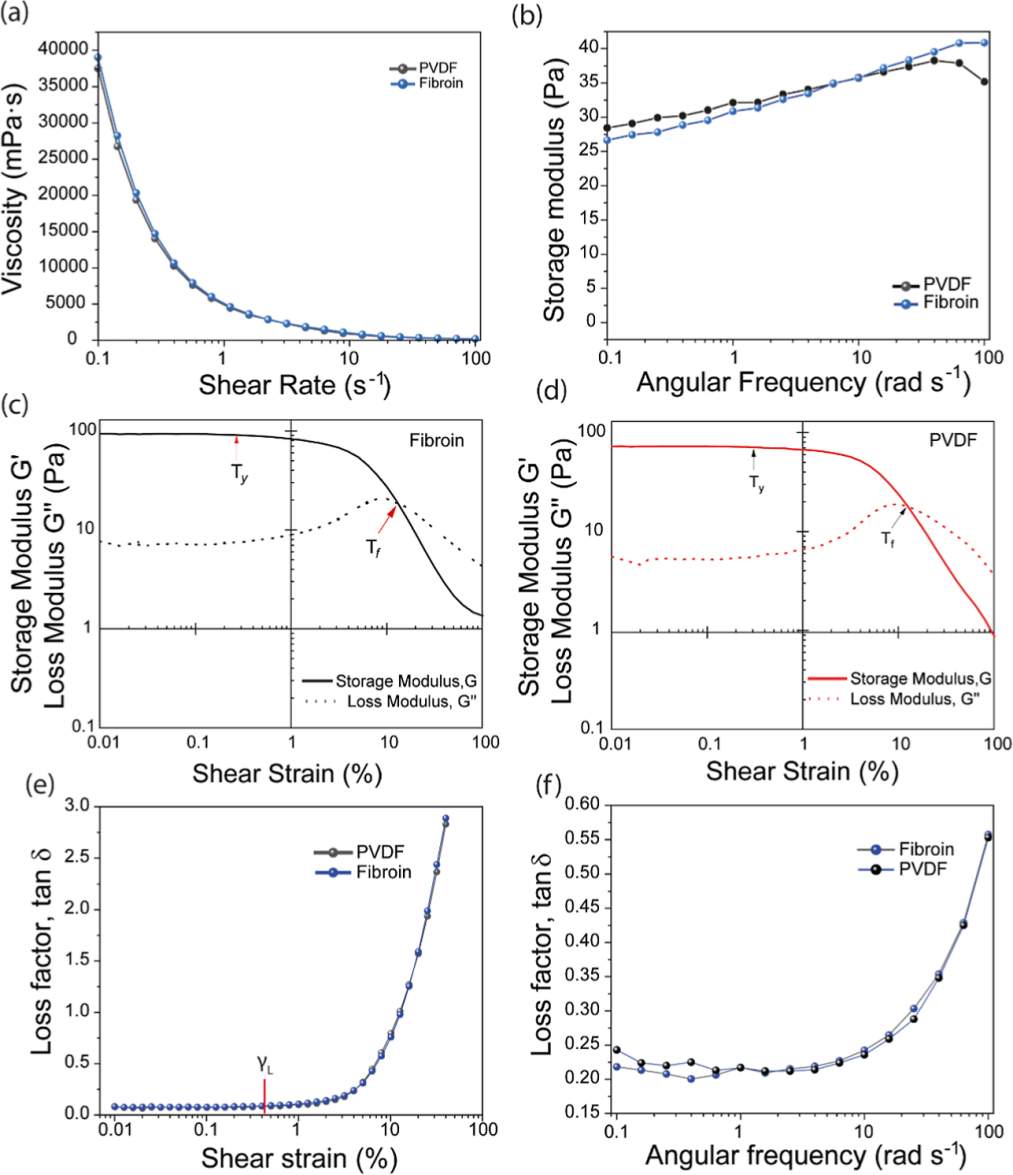
Rheology analysis of fibroin and PVDF binder slurries. (a) Viscosity vs shear rate; (b) storage modulus vs angular frequency; (c,d) storage modulus (*Ǵ*) and loss modulus (*G*″) vs shear strain for fibroin and PVDF, respectively; and (e) loss factor, tan δ, as a function of shear strain of fibroin and PVDF slurries.

Further insights into the structural integrity and particle dispersion within the slurries are provided by the storage modulus versus angular frequency data, presented in Figure 7b. The storage modulus, indicative of the slurry’s carbon-binder network and particle dispersion efficacy, was measured to be 26.65 Pa for fibroin and 28.47 Pa for PVDF at an angular frequency of 0.1 rad s^–1^, suggesting a marginally better particle dispersion stability in PVDF-based slurries. Notably, at elevated frequencies, the storage modulus for PVDF exhibits a pronounced decline, attributed to the disintegration of the carbon-binder network under substantial force, whereas fibroin maintains stability, implying that fibroin slurries can be effectively coated at higher speeds.^43,44^

Comparative analysis of the storage and loss moduli versus shear stress for fibroin and PVDF (Figure 7c,d) reveals that at low-strain regions, the storage modulus (*G*′) surpasses the loss modulus (*G*″), indicating a predominantly elastic response for both slurry types.^45^ The yield point (*T*_y_), establishing the end of the linear viscosity regime, was identified at 0.304 for fibroin and 0.318 for PVDF, with both materials exhibiting an identical flow point (*T*_f_) of 12.7. The flow transition index (*T*_y_/*T*_f_), a metric for assessing the slurry’s propensity for brittle fracture,^46^ was calculated to be 0.0239 for fibroin and 0.0250 for PVDF, suggesting comparable brittleness and “spread-ability” between fibroin and the benchmark PVDF binder. The loss factor (tan δ = *G*″/*G*′), as a function of shear strain, is detailed in Figure 7e, with γ_L_ denoting the boundary of the linear viscoelastic region. Both fibroin and PVDF slurries showcased similar γ_L_ values, indicative of analogous structural robustness and a consistent tendency to maintain a cohesive three-dimensional network structure.^47^ Notably, fibroin exhibited a lower loss factor (tan δ = 0.218) compared to PVDF (tan δ = 0.243) at the lowest angular frequency (Figure 4f), signifying enhanced particle dispersion stability in fibroin-based slurries.^48^

Silk fibroin is the lightest among natural fibers, with tensile strength greater than many natural fibers, and is stronger than steel wire of equal thickness. Fibroin’s mechanical properties are mainly derived from the hierarchical composition and molecular structure of the fibers. The strongly interconnected nanofibrils prevent slipping when subjected to mechanical stress.^49,50^ This unique combination of strength and toughness makes it a robust binder material. To analyze the impact of the fibroin binder on the mechanical properties of the electrode, we carried out tensile strength measurements on the electrode fabricated with fibroin and compared it to the PVDF electrode (Figure S12). We can see in Figure S12a that the maximum force needed for the fibroin electrode to fracture was 53 N, while the PVDF electrode required 48.11 N. A higher maximum force before failure for fibroin reflects higher strength and stronger cohesive forces and adhesion to the substrate than PVDF.^51^ Fibroin electrodes exhibit a slightly lower Young’s modulus (0.206 MPa, more flexible), Figure S12b, compared to electrodes containing PVDF (0.214 MPa). The lower Young’s modulus of the fibroin electrode allows it to absorb expansion more effectively than PVDF, thereby minimizing cracks and delamination during operation.^52^ We also conducted tensile strength measurements on PVDF and fibroin films (Figure S12c); the film obtained from fibroin was highly stiff and brittle, while the film obtained from PVDF was flexible. The Young’s modulus of the fibroin film was measured at 0.204 MPa, while the PVDF film had a Young’s modulus of only 0.074 MPa. A higher Young’s modulus in fibroin film correlates with its stiffness and resistance to deformation. Unlike PVDF, which exhibited plastic deformation due to its flexibility, the fibroin film did not show any plastic deformation because of its high stiffness. It is to be noted that the tensile strength measurement on the binder films is largely dependent on the film properties. Nevertheless, the fibroin electrode demonstrates better cohesion and adhesion while maintaining a lower Young’s modulus, highlighting its advantages as an electrode binder. This comprehensive rheological assessment underscores the potential of fibroin as a viable, aqueous, drop-in alternative to the conventional PVDF binder in electrode slurry formulations, offering identical performance characteristics.

Next, to further highlight industrial viability, we conducted an evaluation of the effectiveness of fibroin as a binder in a more commercially relevant pouch cell format (3.6 mAh). The electrodes used were 25 × 14 mm in size (an image of the pouch cell is shown in Figure S13). Fabrication details are provided in the experimental section. The charge–discharge profile in Figure 8a, at a C/20 rate, shows that the cell achieves a capacity of 1634 mAh g_(S)_^–1^, which is close to the theoretical maximum. The pouch cell configuration enhances material utilization and wetting, resulting in a significantly increased capacity compared to coin cells.^53^ We also examined the durability of the fibroin electrode by subjecting it to 100 charge–discharge cycles at a rate of C/5. The results are shown in Figure 8b, with the pouch cell starting at a capacity of 990 mAh g_(S)_^–1^, which decreased to 795 mAh g_(S)_^–1^ after the 100th cycle, a retention rate of 80%. Throughout cycling, the cell maintained an average Coulombic efficiency of 97%.

**Figure 8:**
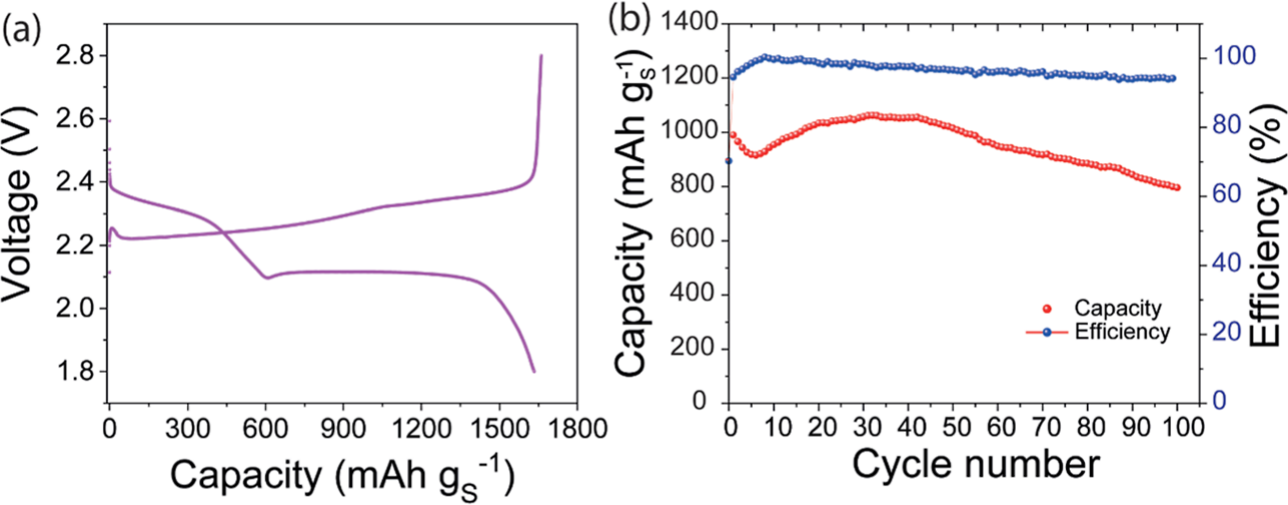
Fibroin pouch cell electrochemical assessment. (a) Charge–discharge at C/20 and (b) cycling data of a pouch cell assembled with fibroin binder at a rate of C/5.

This extensive analysis highlights the potential of fibroin as an effective binder and provides valuable insights into its practical applications in improving the performance and stability of Li–S batteries.

Finally, as highlighted previously, a major downside of the use of PVDF binders is the complexity that they add to the cell recycling process; recyclability will be a key metric for future batteries. PVDF is not only difficult to separate from other electrode components but also potentially highly environmentally toxic if combusted (a common route for electrode material recovery).

In this context, the use of fibroin as a binder in battery electrodes presents significant recycling advantages. Fibroin, a protein, can be simply denatured in the presence of denaturants such as acids or alkalis. Denaturation involves the disruption of weak ionic and hydrogen bonding interactions within the protein, leading to its unfolding.^54^ This characteristic can be leveraged to facilitate the recycling of our battery electrodes, as this weakening in the adhesion interparticle forces within the electrode bulk and the interaction between the electrode material and the current collector can simplify the recovery of all components without necessitating shredding or other energy-intensive processes.

To demonstrate this concept, electrodes employing both PVDF and fibroin binders were subjected to a treatment with acetic acid (5% v/v acetic acid solution) for 30 s to assess the impact of denaturation on the separation of electrode materials from the current collector. As depicted in Figure S14a–c, the active material on the fibroin cell quickly began to detach from the electrode, resulting in the simple recovery of a clean aluminum current collector (Figure S14c), alongside the active materials, which could then be separated and reprocessed. On the contrary, electrodes fabricated with PVDF remained intact and bound after immersing in the acetic acid solution, meaning no materials could be recovered or reused (Figure S14d,e).

## Conclusion

In this work, we explored the application of fibroin as a functional binder in Li–S battery cells, highlighting its capability to stabilize cycling performance by binding polysulfides within the positive electrode and preventing the polysulfide shuttle effect. Comparative studies have revealed that fibroin-based electrodes can deliver near theoretical capacities of 1634 mAh g_(S)_^–1^ at C/20 in pouch cells and maintain 80% capacity after 100 cycles at C/5. Cells containing the fibroin binder are shown to outperform cells fabricated with the conventional PVDF binder in terms of cycling stability, owing to limiting the polysulfide shuttle. Rheological analysis of electrode slurries demonstrated similar shear-thinning behaviors for fibroin and PVDF, though fibroin exhibited slightly better particle dispersion and stability, especially at higher frequencies.

The use of in situ and operando techniques further confirmed the superior performance of fibroin. X-ray CT results indicated that fibroin maintains consistent positive electrode thickness across cycles, reducing active material loss compared with PVDF cells, which showed significant positive electrode shrinkage and lesser negative electrode changes. Optical fluorescence measurements supported these findings, showing reduced polysulfide fluctuations and shuttle effects in fibroin cells across multiple cycles. Collectively these findings, along with the proof-of-concept electrode recycling methodology, position fibroin as a strategic choice for enhancing the sustainability of battery manufacturing and end-of-life management. This work lays a foundational step for future research and application of bioderived materials in advanced battery technologies, potentially reducing the environmental footprint of energy storage systems.

## Supplementary Material



## Notes

Protocols for fluorescence microscopy and results, repeats of charge–discharge and cycling data, cyclic voltammograms at different scan rates, contact angle measurements, EDAX data, tensile strength measurements, XCT tomograms, images of electrode recycling and pouch cell, and table comparing results of this work with literature reports (PDF)
