# Supplementary material for: Fibroin: A Multi-Functional Bio-Derived Binder for Lithium–Sulfur Batteries
Source: ACS Sustain Chem Eng. 2025 Aug 22;13(34):13726–39. doi: 10.1021/acssuschemeng.5c01291 (PMC12406479; doi:10.1021/acssuschemeng.5c01291)
Supplement: Supplementary file 1 [file sc5c01291_si_001.pdf]

Supporting information

Fibroin: A Multi-Functional Bio-Derived Binder for Lithium-Sulfur Batteries

Yiming Guo[†,‡,#], *Roby Soni*[†,‡,□,#]*, *Kofi Coke*[†], *James B. Robinson*[†,‡,□], *Francesco Iacoviello*[†], *Robert S. Young*[†], *Rhodri Jervis*[†,‡,□], *Paul R. Shearing*[ƨ,□], *Thomas S. Miller*[†,‡,□]*

[†]Electrochemical Innovation Lab, Department of Chemical Engineering, University College London, Torrington Place, London WC1E 7JE, U.K.

[‡]Advanced Propulsion Lab, Marshgate, University College London, 7 Sidings Street, London E20 2AE, U.K.

[□]The Faraday Institution, Quad One, Becquerel Avenue, Harwell Campus, Didcot OX11 ORA, U.K.

[ƨ] The ZERO Institute, University of Oxford, Holywell House, Osney Mead, Oxford, OX2 0ES

[#]Equal contribution

Corresponding Authors

Thomas Miller − Electrochemical Innovation Lab, Department of Chemical Engineering, University College London, Torrington Place, London WC1E 7JE, U.K.
Email: t.miller@ucl.ac.uk

Roby Soni − Electrochemical Innovation Lab, Department of Chemical Engineering, University College London, Torrington Place, London WC1E 7JE, U.K.
Email: roby.soni@ucl.ac.uk

(a)

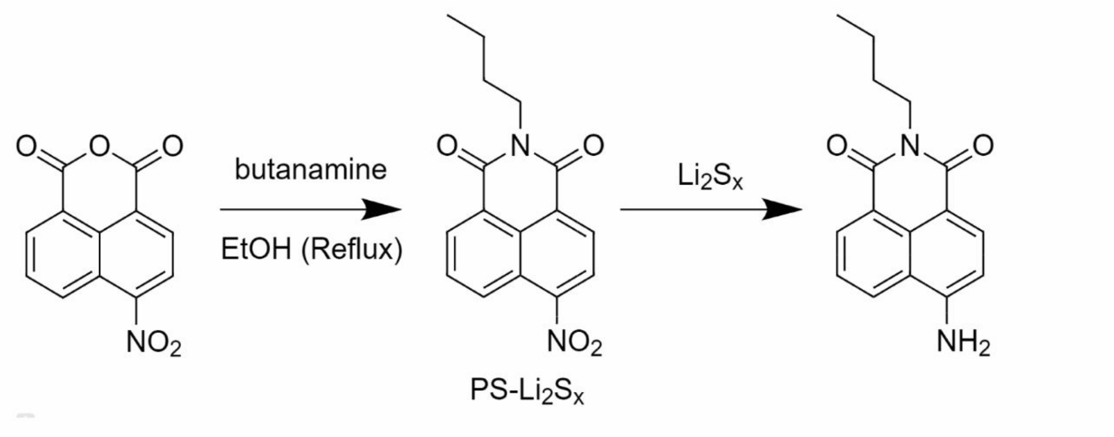

(b)

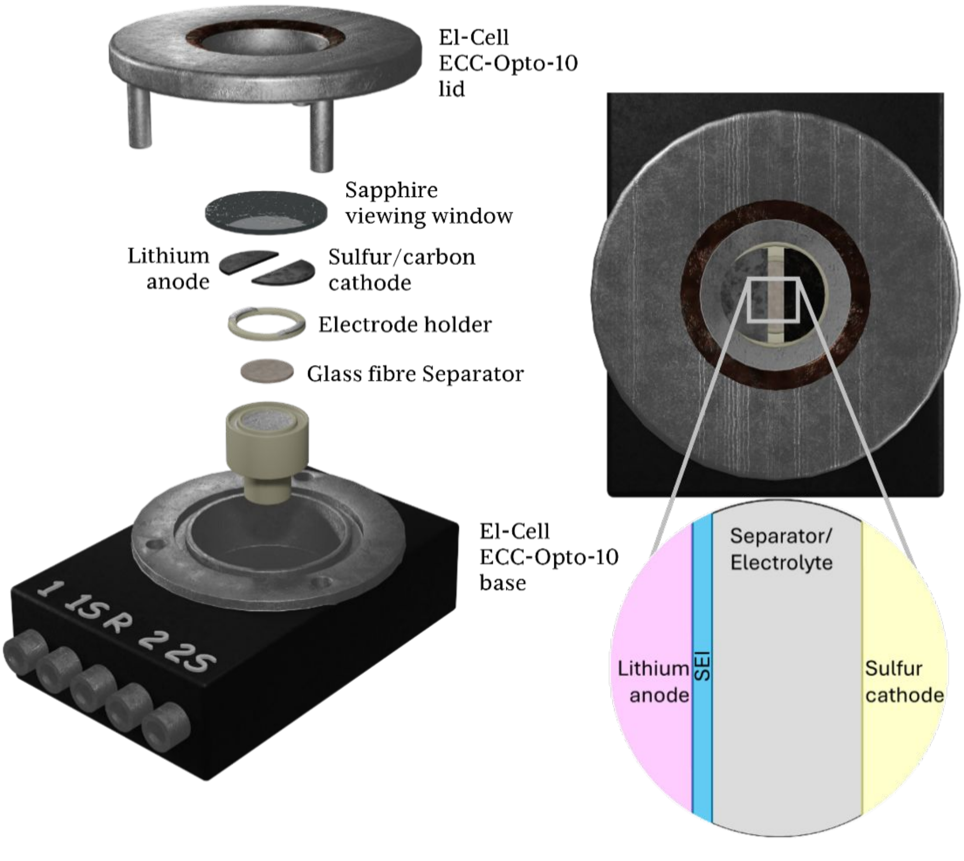

Figure S1. (a) Reaction scheme for the synthesis of the polysulfide sensitive fluorescent dye PS-Li_2S_x. The subsequent reaction of the dye with lithium polysulfides is also given. (b) Blown up schematic of the EL-Cell ECC-Opto-Std, showing the arrangement of semi-circular electrodes under the viewing window. Depiction of the view through the viewing window of the constructed optical microscopy cell. The two electrodes are clearly framed alongside a region of electrolyte. The expansion shows the approximate area imaged by the fluorescence microscope during the experiments.

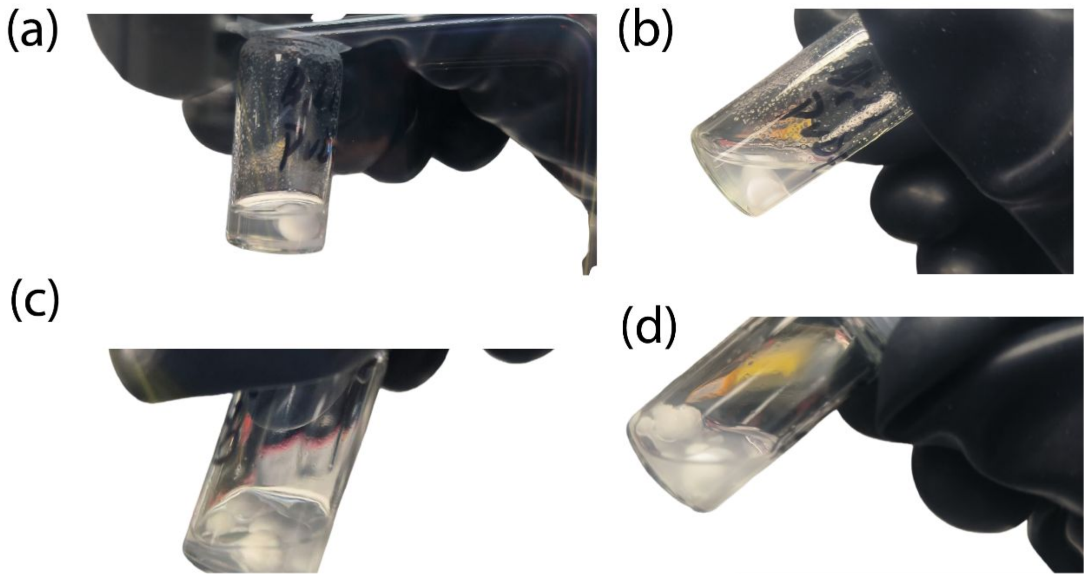

Figure S2. Solubility test. (a) PVDF in DOL/DME mixture after 1 minute. (b) PVDF in DOL/DME mixture after 24 hr. (c) Fibroin DOL/DME mixture after 1 minute. (d) Fibroin in DOL/DME mixture after 24 hr.

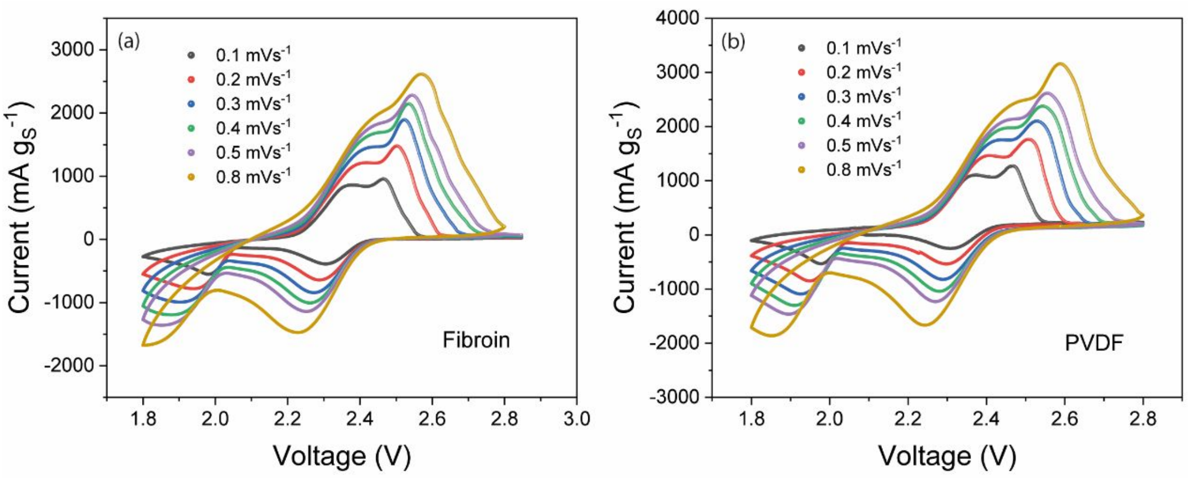

Figure S3. Cyclic voltammograms of (a) Fibroin and (b) PVDF cells at different scan rates.

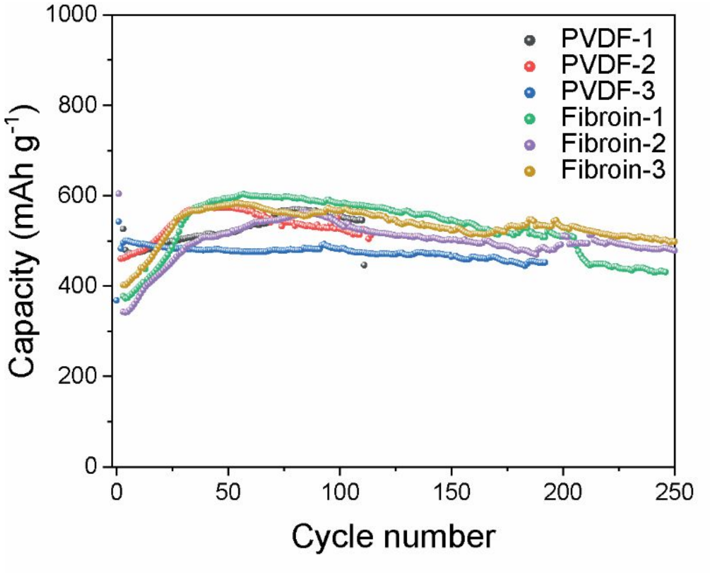

Figure S4. Stability tests of fibroin and PVDF cells carried out at C/5 rate.

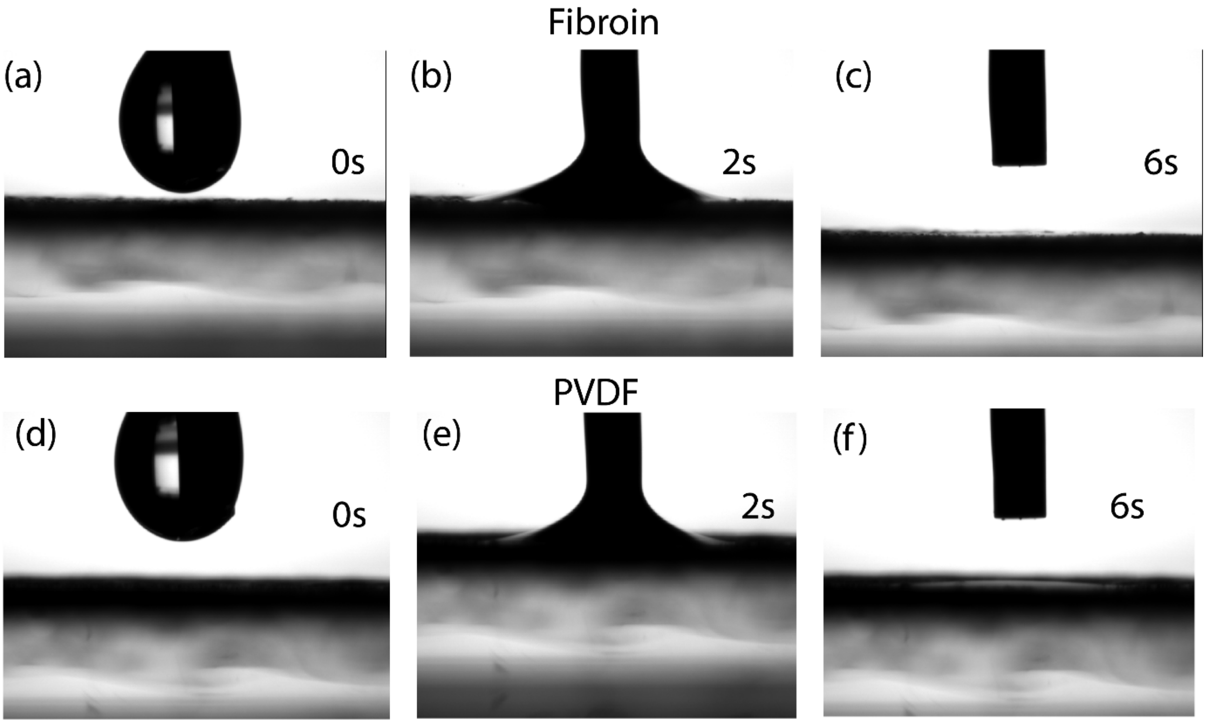

Figure S5. Contact angle measurement. (a-b) show electrolyte contact angel measurement for electrode containing fibroin binder; and (d-f) are the electrolyte contact angel measurement images for PDVF electrodes.

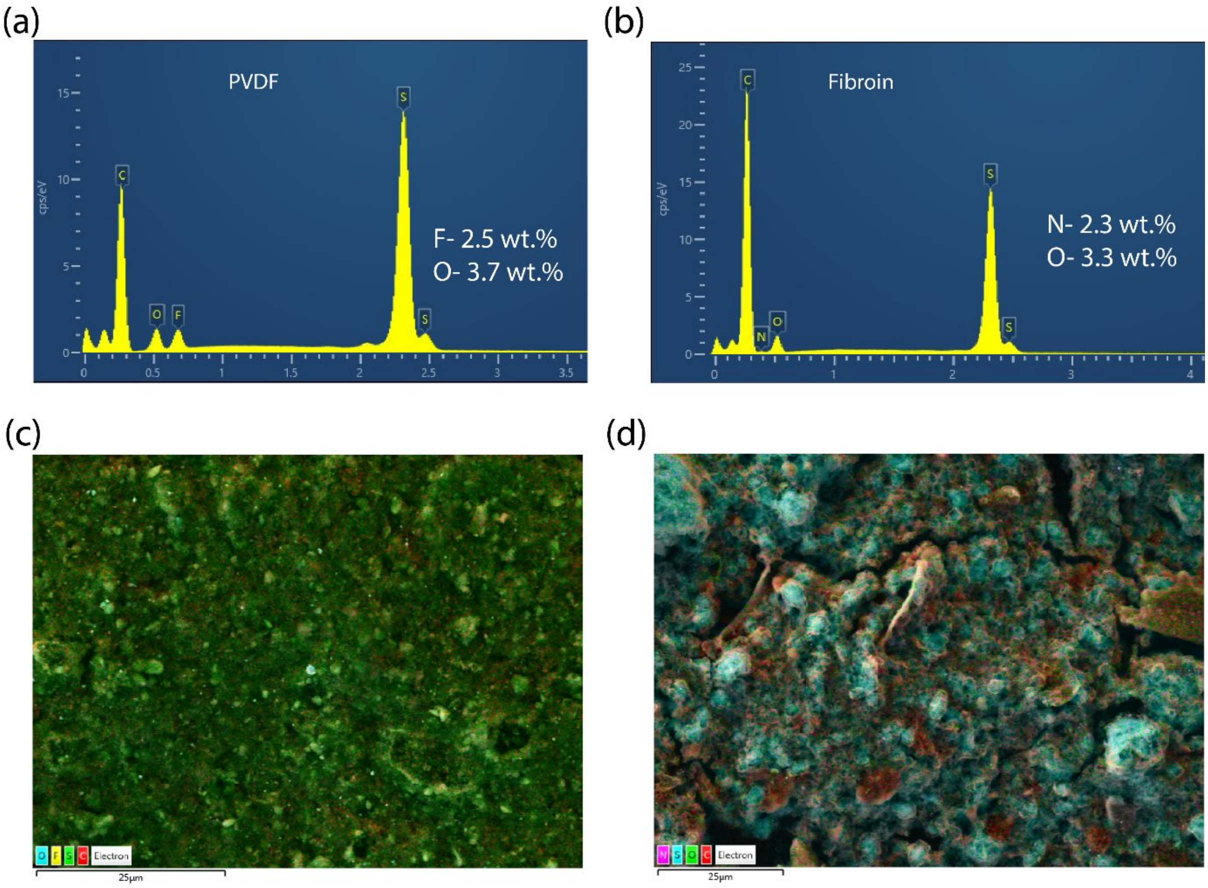

Figure S6. EDAX data. (a) PVDF binder electrode; (b) Fibroin binder electrode; (b) PVDF electrode element overlay; (d) Fibroin electrode element overlay.

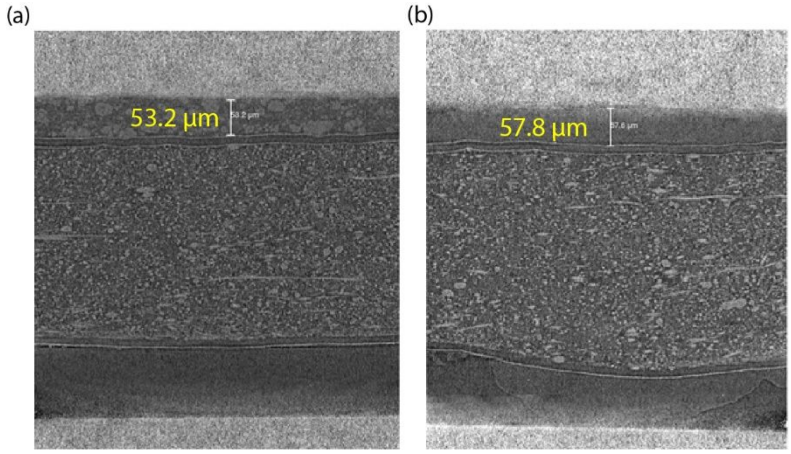

Figure S7. Figure showing increase in thickness of cathode fabricated with fibroin binder. (a) Pristine cathode; (b) Cathode after discharge

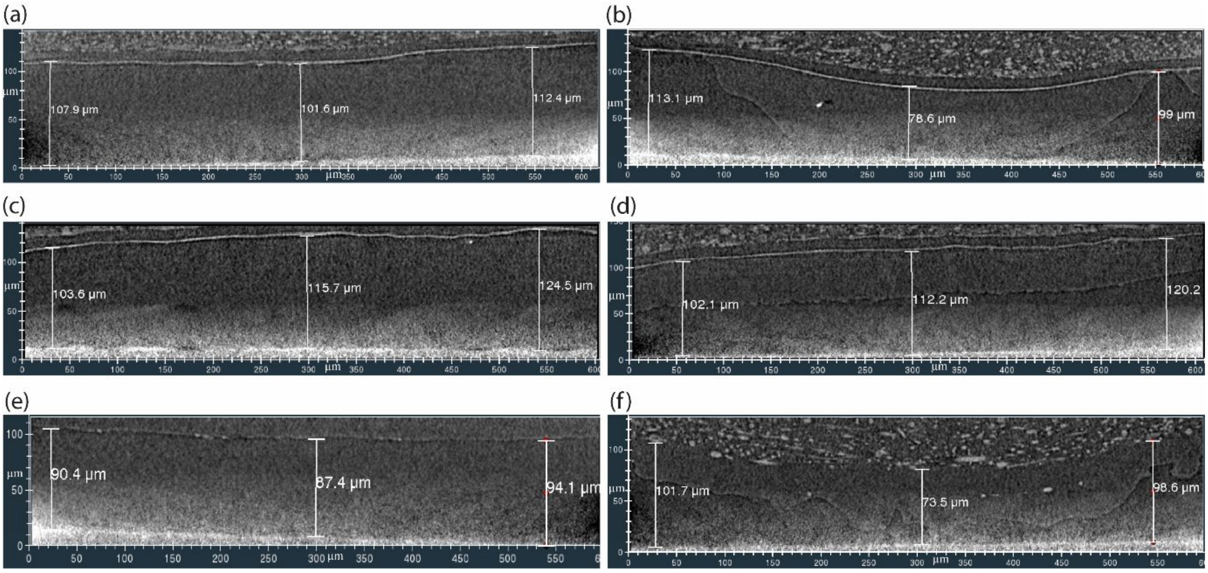

Figure S8. X-ray computed tomography. (a) Lithium of the pristine fibroin cell; (b) Discharged state of Lithium shown in (a); (c) and (d) lithium in pristine state of PVDF cell and discharged state, respectively; (e) and (f) Show the comparison of lithium in the pristine and discharge state of second PVDF cell, respectively.

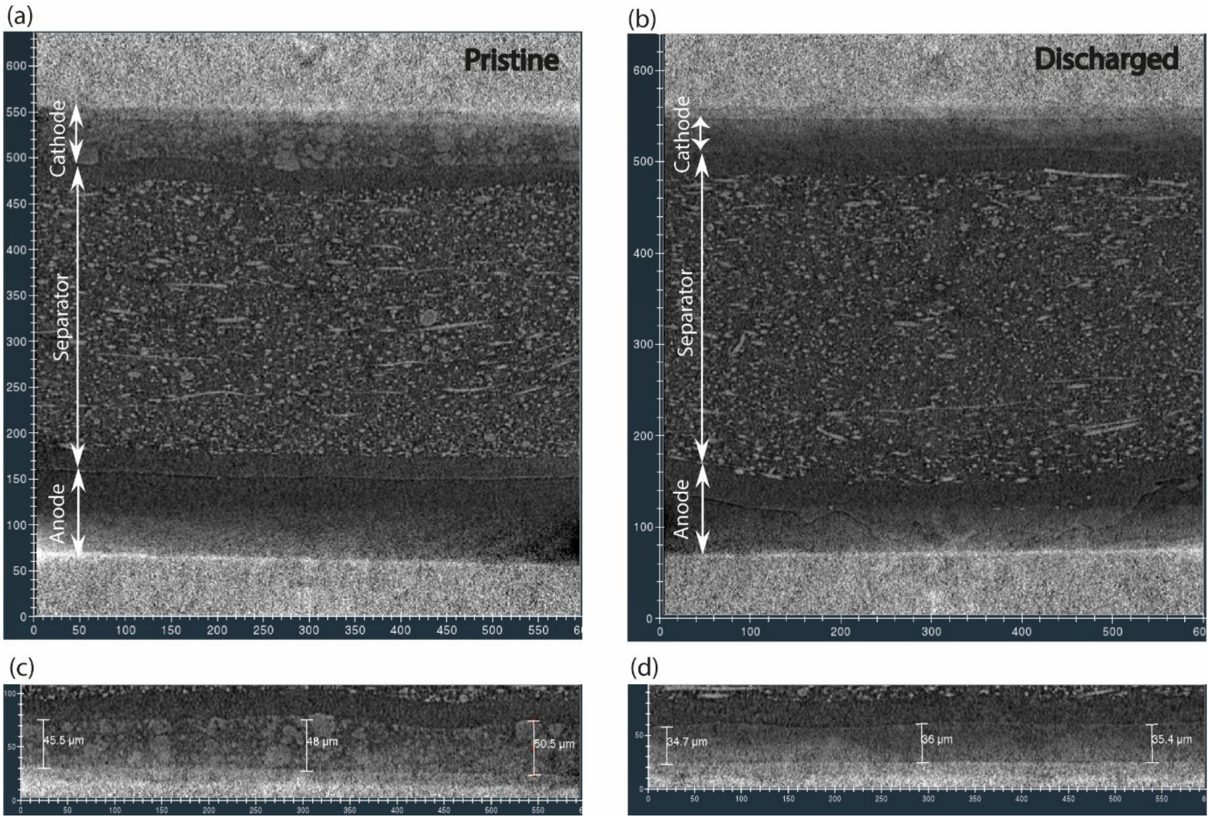

Figure S9. (a) and (b) Tomograms of PVDF cell in the pristine and discharged state, respectively; (c) and (d) Tomograms of cathode in pristine and discharge state, respectively, extracted from (a) and (b).

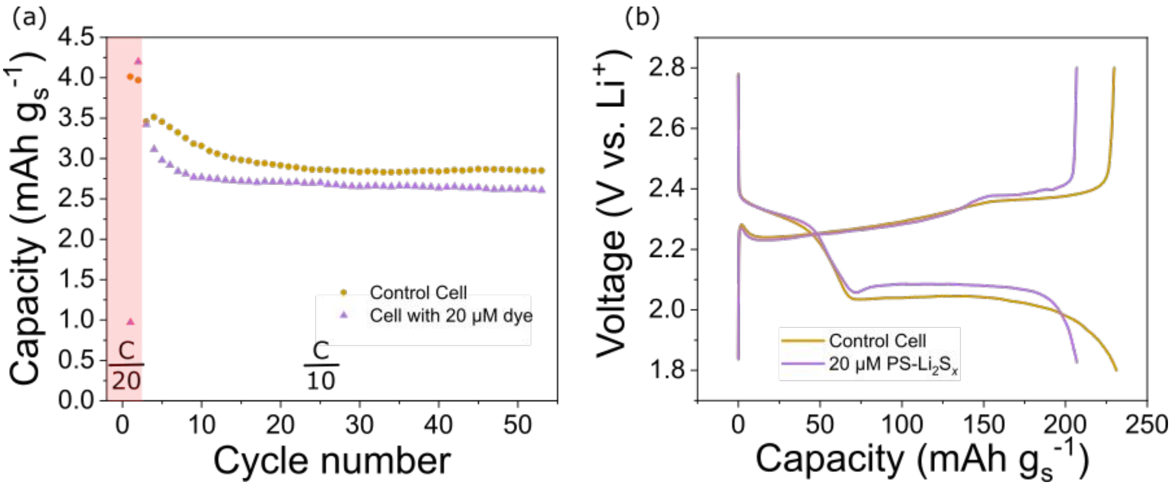

Figure S10. For an Li-S cell using a 0.8 wt% fibroin electrolyte additive, both with and without the addition of 20 µM PS-Li_2S_x, (a) specific discharge capacity (sulfur mass) against cycle number for 53 cycles, with 2 formation cycles at C/20 and a further 50 at C/10 (b) cycling data showing specific capacity (sulfur mass) taken during the fifth cycles at C/10.

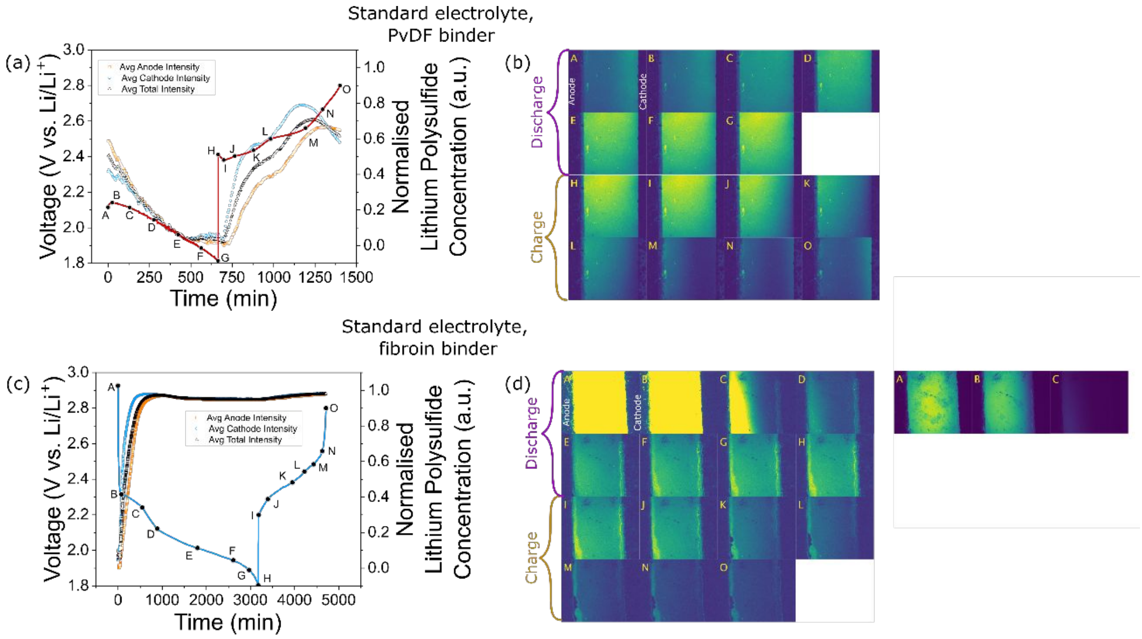

Figure S11. Cycling data with normalised polysulfide concentration (a, c) and optical fluorescence imaging (b, d) taken during the first cycle of an operando study of the electrolyte of a Li-S cell, with 20 µM polysulfide sensitive PS-Li_2S_x fluorescent dye. The cells utilised a PvDF binder and standard electrolyte (a, b) and a fibroin binder and standard electrolyte (c, d).

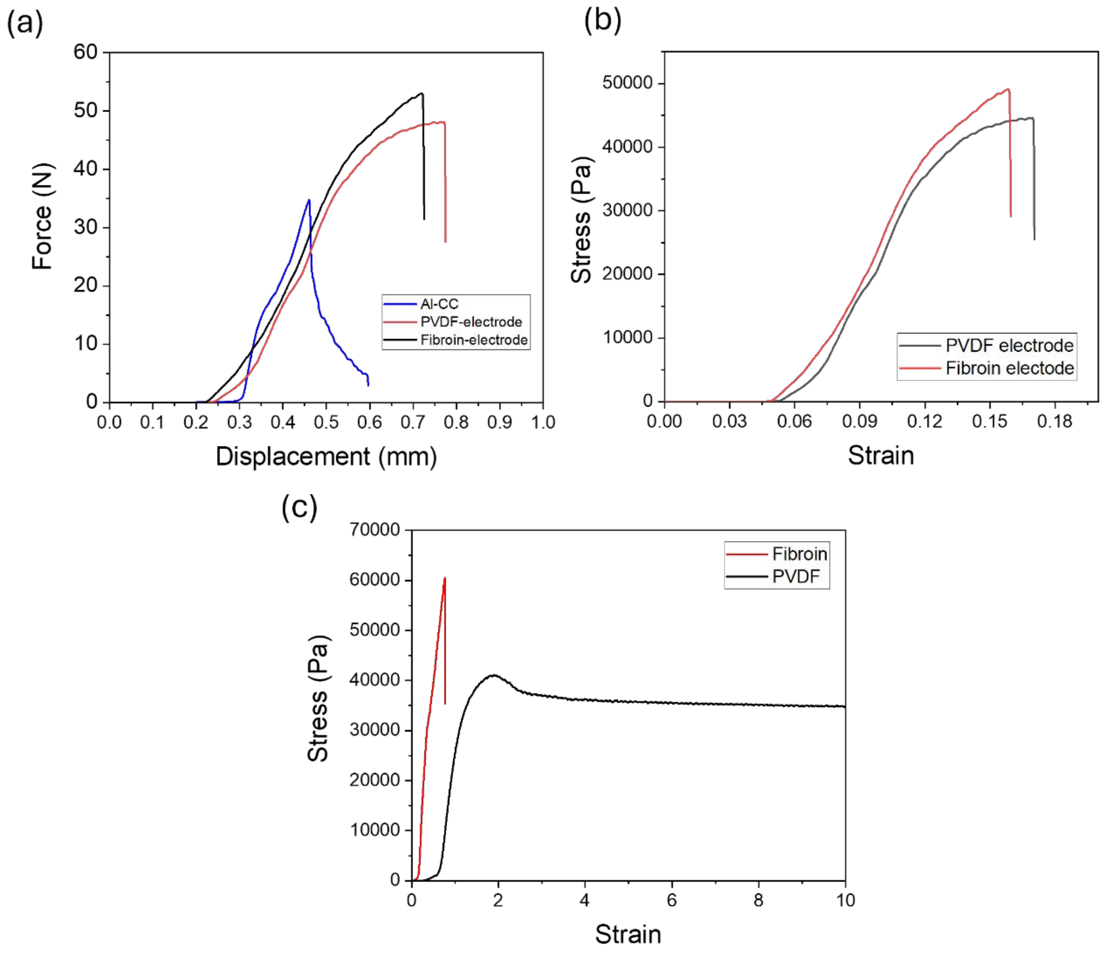

Figure S12. Tensile strength measurement. (a) Force-displacement curves of electrode fabricated with fibroin and PVDF; (b) and (c) Stress-strain curves of electrodes and self-standing binder films, respectively.

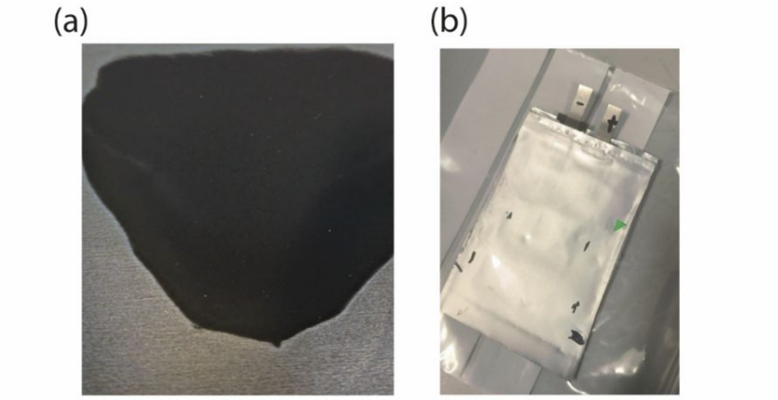

Figure S13. (a) Image of the electrode prepared with Fibroin binder; (b) Photograph of Fibroin pouch cell.

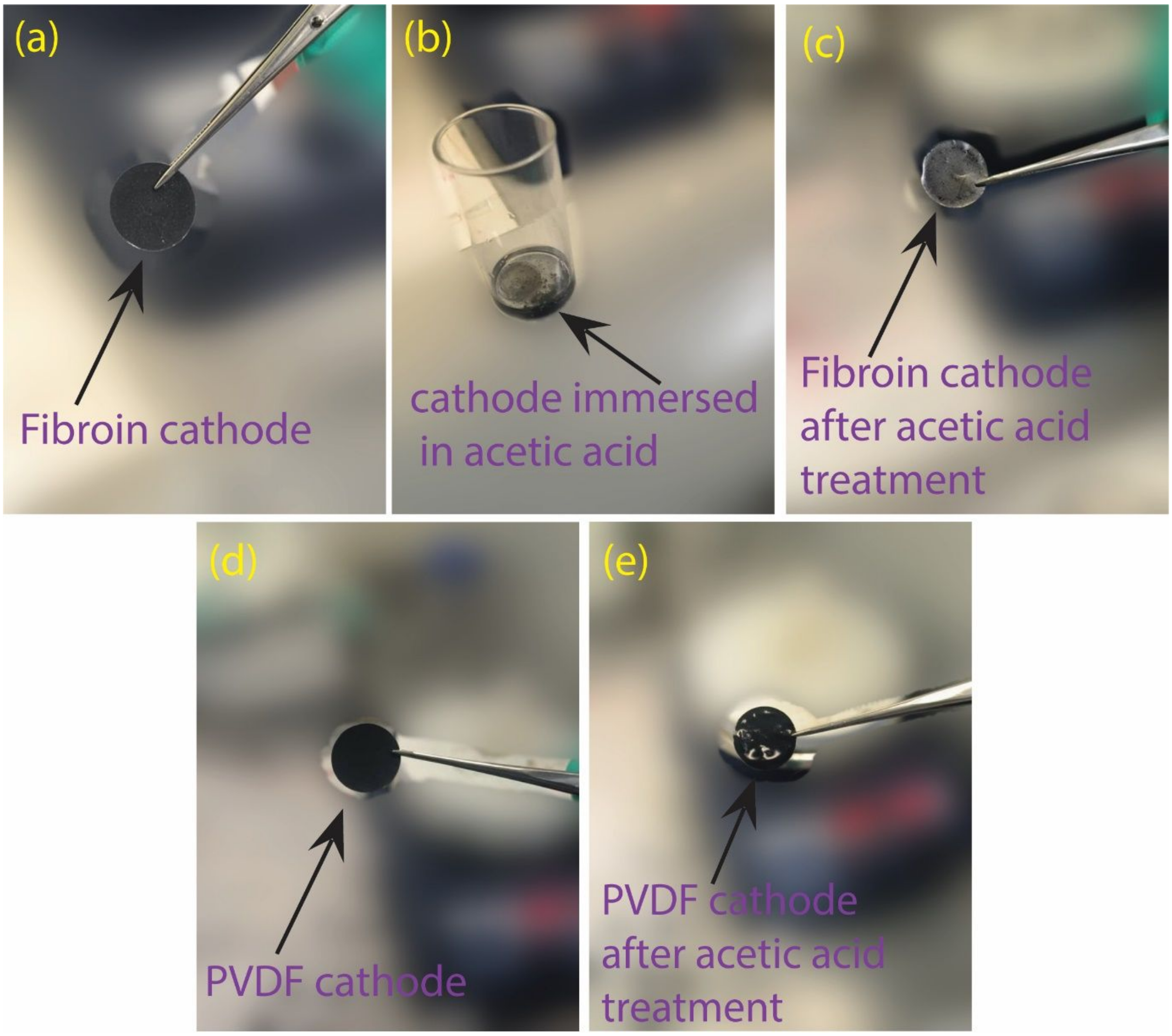

Figure S14. Electrode recycling demonstration. (a) Fibroin electrode; (b) Show detached electrode materials from the current collector after dipping fibroin electrode (a) in 5% v/v acetic acid solution; (c) Recovered current collector from the electrode shown in (a); (d) and (e) Show the electrode fabricated with PVDF binder before treating with acetic acid and after acid treatment, respectively.

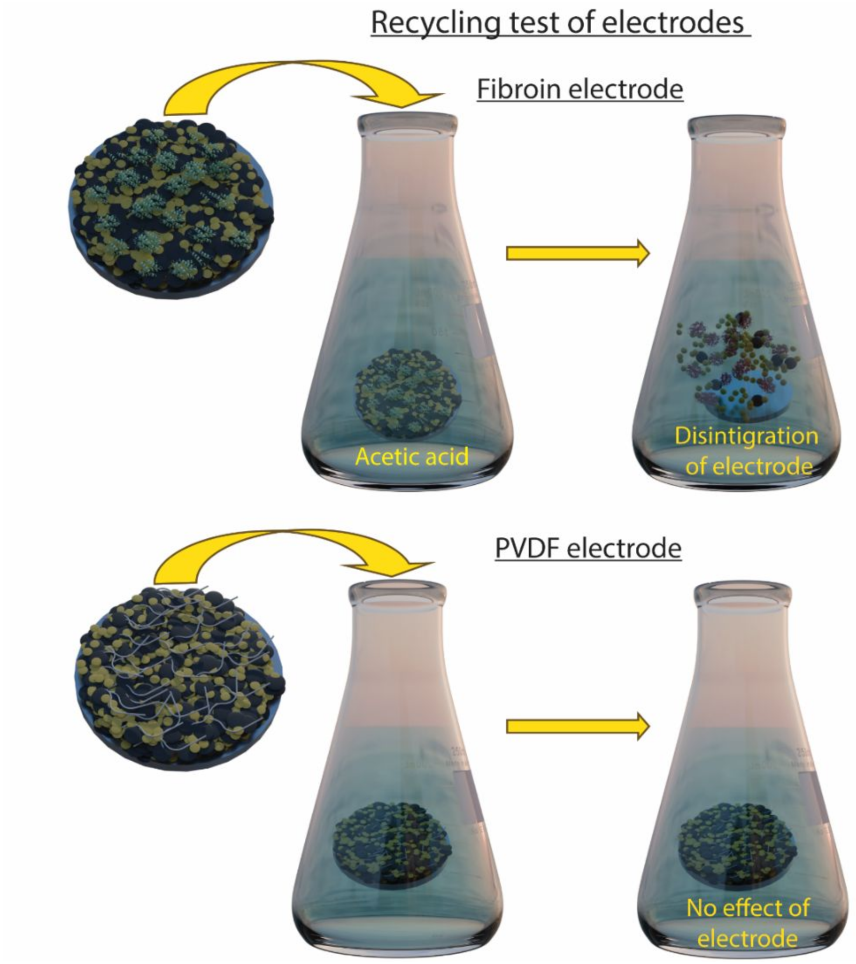

Figure S15. Schematic showing the ease of recycling of fibroin electrode by denaturing agent.

Table S1. The values of the equivalent circuit parameters obtained from EIS fitting

	R_{dl}	R_{SEI}	R_{CT}	W_{diff}
**Fibroin cell**	1.38 Ω	6.87	2.24	1.006
**PVDF cell**	2.71 Ω	4.35	3.92	1.268

Table S2. Comparison of cell characteristics and performance of various organic binder reported in literature with fibroin binder in Li-S cells.

S. No.	Binder	Binder content (wt. %)	Cathode composition, Sulfur wt. % and sulfur loading in electrode	Performance	Reference

1	Sodium alginate	10	Carbon black, 55 wt.% S, 1.5 mg cm^{-2} S	508 mAh g^{-1} at 0.2 C, capacity retention of 74.7% after 50 cycles	[1]
2	Chitosan	10	Multi-walled carbon nanotube, 60 wt. % S, 10 mg cm^{-2} S	1050 mAh g^{-1} at 0.3 C, capacity retention of 91% after 50 cycles	[2]
3	Gelatin	8	Acetylene black, 63 wt. % S, 2 mg cm^{-2} S	463 mAh g^{-1} at 0.2 C, capacity retention of 61.0% after 100 cycles	[3]
4	PGA	7	Acetylene black, 70 wt. % S, 1.2-1.5 mg cm^{-2} S	727 mAh g^{-1} at 0.2 C, capacity retention of 70.2% after 100 cycles	[4]
5	Gum Arabic	20	Carbon black, 44 wt. % S, 0.66-1 mg cm^{-2} S	1090 mAh g^{-1} at 0.2 C, capacity retention of 78.6% after 50 cycles	[5]
6	Guar Gum	15	Acetylene black, 60 wt. % S, 0.6-0.7 mg cm^{-2} S	777 mAh g^{-1} at 0.2 C, capacity retention of 86.3% after 150 cycles	[6]
7.	Amylopectin	14	GO, 62 wt. % S, 2-6 mg cm^{-2} S	441 mAh g^{-1} at 0.3 C, capacity retention of 68% after 175 cycles	[7]
8.	Carbonyl-β-cyclodextrin	10	SPAN, 36 wt. % S, 3 mg cm^{-2} S	1456 mAh g^{-1} at 0.2 C, capacity retention of 94.4% after 50 cycles	[8]
9.	Starch	10	Super P, 70 wt. % S, 1 mg cm^{-2} S	594.3 mAh g^{-1} at 0.2 C, capacity retention of 94% after 100 cycles	[9]
10.	CMC: SBR	10	Carbon black, 60wt. % S, areal sulfur loading NA	580 mAh g^{-1} at 0.06 C, capacity retention of 66.7% after 60 cycles	[10]
11.	PEO	10	Ketjen black, 60 wt. % S, 0.5 mg cm^{-2} S	800 mAh g^{-1} at 0.08 C, capacity retention of 74.7% after 30 cycles	[11]
12.	PVP	5	Carbon black, Li_2S, 42 wt. % S, 1.4 mg cm^{-2} S	714 mAh g^{-1} at 0.2 C, capacity retention of 94% after 100 cycles	[12]
13.	LA132	5	Active carbon, 35 wt. % S, 2 mg cm^{-2} S	470 mAh g^{-1} at 0.5 C, capacity retention of 52.1% after 100 cycles	[13]
14.	PAA	10	Carbon black, 60 wt. % S, 1.5 mg cm^{-2} S	325 mAh g^{-1} at 0.2 C, capacity retention of 42.9% after 50 cycles	[14]
15.	Fibroin	10	C65,70 wt. % S, 3-3.5 mg cm^{-2} S	500-600 mAh g^{-1} at 0.2 C, 80% retention after 200 cycles	This work

References

(1) Bao, W.; Zhang, Z.; Gan, Y.; Wang, X.; Lia, J. Enhanced Cyclability of Sulfur Cathodes in Lithium-Sulfur Batteries with Na-Alginate as a Binder. *Journal of Energy Chemistry* 2013, *22* (5), 790–794. https://doi.org/10.1016/S2095-4956(13)60105-9.

(2) Kim, H. M.; Sun, H. H.; Belharouak, I.; Manthiram, A.; Sun, Y. K. An Alternative Approach to Enhance the Performance of High Sulfur-Loading Electrodes for Li-S Batteries. *ACS Energy Lett* 2016, *1* (1), 136–141. https://doi.org/10.1021/ACSENERGYLETT.6B00104/ASSET/IMAGES/LARGE/NZ-2016-001047_0004.JPEG.

(3) Liu, N.; Huang, B.; Wang, W.; Shao, H.; Li, C.; Zhang, H.; Wang, A.; Yuan, K.; Huang, Y. Modified Separator Using Thin Carbon Layer Obtained from Its Cathode for Advanced Lithium Sulfur Batteries. *ACS Appl Mater Interfaces* 2016, *8* (25), 16101–16107. https://doi.org/10.1021/ACSAMI.6B04418/ASSET/IMAGES/LARGE/AM-2016-04418M_0005.JPEG.

(4) Qiu, T.; Shao, H.; Wang, W.; Zhang, H.; Wang, A.; Feng, Z.; Huang, Y. Development of a γ-Polyglutamic Acid Binder for Cathodes with High Mass Fraction of Sulfur. *RSC Adv* 2016, *6* (104), 102626–102633. https://doi.org/10.1039/C6RA20504F.

(5) Li, G.; Ling, M.; Ye, Y.; Li, Z.; Guo, J.; Yao, Y.; Zhu, J.; Lin, Z.; Zhang, S. Acacia Senegal-Inspired Bifunctional Binder for Longevity of Lithium-Sulfur Batteries. *Adv Energy Mater* 2015, *5* (21), 1500878. https://doi.org/10.1002/AENM.201500878;PAGEGROUP:STRING:PUBLICATION.

(6) Lu, Y. Q.; Li, J. T.; Peng, X. X.; Zhang, T.; Deng, Y. P.; Wu, Z. Y.; Deng, L.; Huang, L.; Zhou, X. D.; Sun, S. G. Achieving High Capacity Retention in Lithium-Sulfur Batteries with an Aqueous Binder. *Electrochem commun* 2016, *72*, 79–82. https://doi.org/10.1016/J.ELECOM.2016.09.004.

(7) Zhou, W.; Chen, H.; Yu, Y.; Wang, D.; Cui, Z.; Disalvo, F. J.; Abruña, H. D. Amylopectin Wrapped Graphene Oxide/Sulfur for Improved Cyclability of Lithium-Sulfur Battery. *ACS Nano* 2013, *7* (10), 8801–8808. https://doi.org/10.1021/NN403237B/SUPPL_FILE/NN403237B_SI_001.PDF.

(8) Wang, J.; Yao, Z.; Monroe, C. W.; Yang, J.; Nuli, Y. Carbonyl-β-Cyclodextrin as a Novel Binder for Sulfur Composite Cathodes in Rechargeable Lithium Batteries. *Adv Funct Mater* 2013, *23* (9), 1194–1201. https://doi.org/10.1002/ADFM.201201847.

(9) Duan, X.; Han, Y.; Li, Y.; Chen, Y. Improved Capacity Retention of Low Cost Sulfur Cathodes Enabled by a Novel Starch Binder Derived from Food. *RSC Adv* 2014, *4* (105), 60995–61000. https://doi.org/10.1039/C4RA10953H.

(10) He, M.; Yuan, L. X.; Zhang, W. X.; Hu, X. L.; Huang, Y. H. Enhanced Cyclability for Sulfur Cathode Achieved by a Water-Soluble Binder. *Journal of Physical Chemistry C* 2011, *115* (31), 15703–15709.

https://doi.org/10.1021/JP2043416/ASSET/IMAGES/LARGE/JP-2011-043416_0004.JPEG.

(11) Nakazawa, T.; Ikoma, A.; Kido, R.; Ueno, K.; Dokko, K.; Watanabe, M. Effects of Compatibility of Polymer Binders with Solvate Ionic Liquid Electrolytes on Discharge and Charge Reactions of Lithium-Sulfur Batteries. *J Power Sources* 2016, *307*, 746–752. https://doi.org/10.1016/J.JPOWSOUR.2016.01.045.

(12) Seh, Z. W.; Zhang, Q.; Li, W.; Zheng, G.; Yao, H.; Cui, Y. Stable Cycling of Lithium Sulfide Cathodes through Strong Affinity with a Bifunctional Binder. *Chem Sci* 2013, *4* (9), 3673–3677. https://doi.org/10.1039/C3SC51476E.

(13) Pan, J.; Xu, G.; Ding, B.; Han, J.; Dou, H.; Zhang, X. Enhanced Electrochemical Performance of Sulfur Cathodes with a Water-Soluble Binder. *RSC Adv* 2015, *5* (18), 13709–13714. https://doi.org/10.1039/C4RA15303K.

(14) Zhang, Z.; Bao, W.; Lu, H.; Jia, M.; Xie, K.; Lai, Y.; Li, J. Water-Soluble Polyacrylic Acid as a Binder for Sulfur Cathode in Lithium-Sulfur Battery. *ECS Electrochemistry Letters* 2012, *1* (2), A34. https://doi.org/10.1149/2.009202EEL/XML.
